# 9-*O*-Acetylation of sialic acids is catalysed by CASD1 *via* a covalent acetyl-enzyme intermediate

**DOI:** 10.1038/ncomms8673

**Published:** 2015-07-14

**Authors:** Anna-Maria T. Baumann, Mark J. G. Bakkers, Falk F. R. Buettner, Maike Hartmann, Melanie Grove, Martijn A. Langereis, Raoul J. de Groot, Martina Mühlenhoff

**Affiliations:** 1 Institute of Cellular Chemistry, Hannover Medical School, Hannover, D-30623 Germany; 2 Virology Division, Department of Infectious Diseases and Immunology, Faculty of Veterinary Medicine, Utrecht University, Utrecht, 3584 CH The Netherlands

**Keywords:** Biochemistry, Carbohydrates

## Abstract

Sialic acids, terminal sugars of glycoproteins and glycolipids, play important roles in development, cellular recognition processes and host–pathogen interactions. A common modification of sialic acids is 9-*O*-acetylation, which has been implicated in sialoglycan recognition, ganglioside biology, and the survival and drug resistance of acute lymphoblastic leukaemia cells. Despite many functional implications, the molecular basis of 9-*O*-acetylation has remained elusive thus far. Following cellular approaches, including selective gene knockout by CRISPR/Cas genome editing, we here show that *CASD1*—a previously identified human candidate gene—is essential for sialic acid 9-*O*-acetylation. *In vitro* assays with the purified N-terminal luminal domain of CASD1 demonstrate transfer of acetyl groups from acetyl-coenzyme A to CMP-activated sialic acid and formation of a covalent acetyl-enzyme intermediate. Our study provides direct evidence that CASD1 is a sialate *O*-acetyltransferase and serves as key enzyme in the biosynthesis of 9-*O*-acetylated sialoglycans.

**Supplementary information:**

The online version of this article (doi:10.1038/ncomms8673) contains supplementary material, which is available to authorized users.

## Abstract (web summary)

9-*O*-Acetylation is one of the most common modifications of sialic acids, implicated in sialoglycan recognition and ganglioside biology. Here, the authors show that the key enzyme for the biosynthesis of 9-*O*-acetylated sialoglycans is CASD1, which uses CMP-activated sialic acid as acceptor substrate.

**Supplementary information:**

The online version of this article (doi:10.1038/ncomms8673) contains supplementary material, which is available to authorized users.

## Graphical abstract

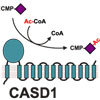

## Introduction

Sialic acids (Sias) are acidic nine-carbon sugars that commonly cap the glycan chains of cell surface glycoproteins and glycolipids. Sialoglycotopes are key determinants for numerous cell recognition events and play fundamental roles in development and host–pathogen interactions^1^. Of note, Sias show a pronounced structural diversity that arises from *N*- and *O*-substitutions of the parent compound neuraminic acid and this diversity is of critical importance for Sia recognition and subsequent cellular processes. For example, the prevalent human Sia 5-*N*-acetylneuraminic acid (Neu5Ac) can be substituted by 9-*O-*acetylation, a modification that affects the binding of several Sia-specific lectins and has functional implications in particular in the immune system^2,3,4,5,6^. 9-*O*-Acetylation impairs host cell binding of influenza A and B viruses, but at the same time creates cellular receptors for influenza C and several nidoviruses^7,8,9^. Another prominent example for the impact of 9-*O*-acetylation is given by CD22, a B-cell-restricted member of the Sia-binding immunoglobulin-type lectin (siglec) family. On binding to α2,6-sialylated *N*-glycans, CD22 regulates B-cell antigen receptor signalling by setting a threshold for receptor activation^10^. *In vitro* data showed that the CD22–ligand interaction is blocked by 9-*O*-acetylation^11^, a process that is *in vivo* tightly controlled by two counteracting enzymatic activities: sialate *O*-acetyltransferase (SOAT) and sialate 9-*O*-acetylesterase (SIAE). Ablation of SIAE function by targeted gene knockout in mice resulted in increased Sia 9-*O*-acetylation and defective CD22 signalling in B-cells and caused defects in peripheral B-cell development and tolerance^12^. Moreover, catalytically defective rare variants of SIAE were associated with autoimmune diseases in humans^13,14^.

Sia *O*-acetylation is also a developmentally regulated modification of gangliosides, implicated in neural precursor cell migration, peripheral nerve regeneration and *Mycobacterium leprae* infection of Schwann cells^15,16,17^. Best studied is the 9-*O-*acetylation of GD3, a disialo ganglioside that acts as a potent inducer of apoptosis on translocation to mitochondrial membranes^18^. 9-*O*-Acetylation of the terminal α2,8-linked Sia residue blocks the pro-apoptotic activity of GD3 and promotes survival of cancer cells^19,20,21^. In acute lymphoblastic leukaemia (ALL), survival and drug resistance of lymphoblasts critically depend on 9-*O-*acetylation, which was found on both GD3 and sialoglycoproteins^22,23^. Enzymatic removal of 9-*O*-acetyl groups from internal and cell surface-bound Sias was lethal to ALL cells, opening new perspectives for therapeutic concepts^23^. The use of monoclonal antibodies recognizing the carbohydrate epitopes of non-*O*-acetylated GD3 (CD60a), 7-*O*-acetylated GD3 (CD60c) or 9-*O*-acetylated GD3 (CD60b) revealed a stimulatory or co-stimulatory effect of anti-CD60c and anti-CD60b antibodies on the proliferation of human lymphocytes and implicated distinct roles of 7-*O*- and 9-*O*-acetylated GD3 during activation and apoptosis of tonsillar B and T lymphocytes^24,25^.

Biochemical studies demonstrated that *O-*acetylation of Sia is a postsynthetic modification that takes place in the Golgi apparatus, probably in concert with Golgi-resident sialyltransferases and coupled with the import of acetyl groups from cytosolic acetyl-coenzyme A (acetyl-CoA), the presumptive acetyl donor^26,27^. However, the genes encoding eukaryotic SOATs have remained unknown. Despite many attempts, expression cloning was not successful^28,29,30^ and isolation of SOAT activity proved difficult, mainly due to an inherent sensitivity to membrane solubilization^31,32,33,34^. Searching for human candidate genes, Arming *et al.*^35^ identified *CASD1* (CAS1 domain containing 1), which shares sequence similarity to *CAS1* (Capsule synthesis 1) of the pathogenic fungus *Cryptococcus neoformans*. Deletion of the fungal gene revealed an essential role in mannose *O-*acetylation of the unique fungal capsular polysaccharide glucuronoxylomannan^36^. By contrast, little is known on the function of human *CASD1*. *CASD1* overexpression as well as short interfering RNA knockdown assays performed in the initial study by Arming *et al.* indicated a role in 7-*O*-acetylation of GD3 (ref. 35). However, studies addressing SOAT function of a given protein by transfection studies proved, so far, difficult due to endogenous SOAT activity in many common mammalian cell lines^29,35,37^, a problem that might have impeded also previous expression cloning approaches.

On the basis of a naturally occurring 9-*O*-Ac-negative cell line and *CASD1* knockout cells generated by CRISPR/Cas-mediated genome editing, we now show that CASD1 is essential for 9-*O-*acetylation of cellular sialoglycoconjugates. Insight into the catalytic mechanism of CASD1 is given by biochemical analysis of a soluble CASD1 variant encompassing the N-terminal catalytic domain. This reveals the formation of a covalent acetyl-enzyme intermediate, which involves S94 placed in a catalytic triad. Finally, we provide direct *in vitro* evidence for SOAT activity by demonstrating CASD1-mediated transfer of acetyl groups from acetyl-CoA to CMP-activated Neu5Ac.

## Results

### Homology modelling and topology of CASD1

**Figure 1:**
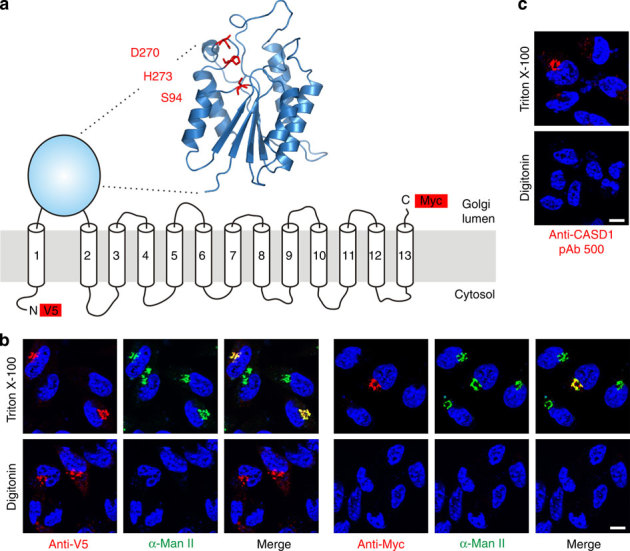
Homology modelling and topology of human CASD1. (**a**) Proposed model of CASD1. The overall architecture of the catalytic domain was predicted by homology modelling using the server Phyre2 and the crystal structure of an isoamyl acetate-hydrolysing esterase from *Saccharomyces cerevisiae* (PDB ID 3mil) as template. The model encompasses residues 83-290 of CASD1 and the predicted catalytic triad is highlighted in red. TMDs are depicted as predicted by the program TMHMM 2.0 (Supplementary Fig. 1). Epitope tags used for the localization of N- and C-terminus are shown as red boxes. (**b**) Orientation of the N- and C-terminus of CASD1. LM-*TK*^−^ cells expressing CASD1 with an N-terminal V5-tag and a C-terminal Myc-tag (V5-CASD1-Myc) were fixed and permeabilized with Triton X-100 (0.2%) or digitonin (5 μg ml^−1^). Cells were stained with anti-V5 or anti-Myc mAb (red) in combination with anti-α-mannosidase II (α-Man II) pAb (green) recognizing the luminal domain of the Golgi marker α-Man II. Nuclei were counterstained with DAPI (blue). (**c**) Orientation of the catalytic domain of CASD1. LM-*TK*^−^ cells expressing CASD1 were permeabilized as described in **b** and stained with pAb 500 directed against the catalytic domain of CASD1. Nuclei were counterstained with DAPI (blue). Scale bar, 10 μm. DAPI, 4,6-diamidino-2-phenylindole; TMD, transmembrane domain.

Human *CASD1* encodes a multimembrane spanning protein of 797 amino acids. For residues 83–290, homology modelling predicted a GDSL/SGNH-like α/β-fold that forms the scaffold for a catalytic triad composed of S94, D270 and H273, with S94 as part of a conserved GDS sequence motif (Fig. 1a). The acronyms of the family name GDSL/SGNH reflect the presence of four invariant residues, namely S in a GDS(L) motif, G located after strand S2, N placed in a GxND motif after strand S3 and H found in a DxxH motif N-terminal to the last helix^38^. However, the N-terminal domain of CASD1 differs from the canonical GDSL/SGNH fold by lacking the conserved residues G and N, and is therefore grouped into pfam family PF13839 (GDSL/SGNH-like acyl-esterase family found in Pmr5 and Cas1p)^39^.

To analyse the subcellular localization and topology of CASD1, a full-length variant, containing an N-terminal V5 and a C-terminal Myc epitope, was expressed in the murine fibroblast cell line LM-*TK*^−^. The orientation of the epitope tags was determined by selective membrane permeabilization and immunofluorescence analysis (Fig. 1b,c). Under conditions that allowed antibody access to all cellular compartments (0.2% Triton X-100), both tags were detectable and showed co-localization with the Golgi marker α-mannosidase II (α-Man II). Selective permeabilization of the plasma membrane with low concentrations of digitonin (5 μg ml^−1^) allowed visualization of the N-terminal V5-tag, but not the C-terminal Myc-tag, demonstrating cytosolic and luminal orientation of N- and C-terminus, respectively. The integrity of the Golgi membrane was demonstrated by parallel staining with an antibody specific for the luminal domain of α-Man II^40^. Staining was observed exclusively in Triton X-100, but not in digitonin-permeabilized cells. Identical results were obtained on staining with an antibody against the SGNH-like domain of CASD1 (pAb 500), demonstrating that this domain faces the Golgi lumen (Fig. 1c). On the basis of these results and on our predictions for the CASD1 transmembrane domains (Supplementary Fig. 1), we propose a topology model encompassing a luminal catalytic domain flanked by one N-terminal and 12 C-terminal transmembrane domains (Fig. 1a).

### CASD1 forms a covalent acetyl-enzyme intermediate

**Figure 2:**
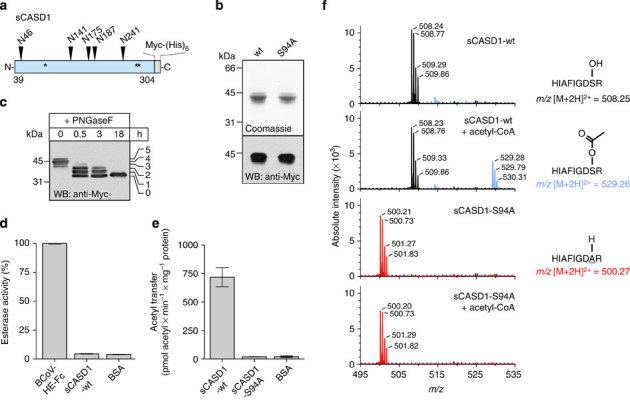
CASD1 forms a covalent acetyl-enzyme intermediate. (**a**) Scheme depicting the soluble CASD1 variant (sCASD1, residues 39-304) used for *in vitro* studies. Predicted *N*-glycosylation sites and putative catalytic residues (S94, D270 and H273) are indicated by triangles and asterisks, respectively. (**b**) Soluble CASD1 variants encompassing the wild-type sequence or the amino-acid exchange S94A were expressed in insect cells and affinity purified from the culture supernatant via the C-terminal hexahistidine tag. Purified proteins were analysed by 12% SDS–PAGE followed by Coomassie staining (upper panel) or western blotting using an anti-Myc mAb (lower panel). (**c**) Analysis of the *N*-glycosylation pattern of sCASD1. Purified sCASD1-wt was analysed before (0 h) and after partial (0.5–3 h) or complete (18 h) Peptide-*N*-glycosidase F (PNGaseF) digest by 12% SDS–PAGE and western blotting using an anti-Myc mAb. Partial removal of *N*-glycans by PNGaseF resulted in five bands with reduced molecular mass, indicating the presence of five *N*-glycans. (**d**) Potential esterase activity of sCASD1-wt was monitored using the acetylesterase substrate pNP-acetate. BCoV-HE-Fc harbouring an active esterase domain was used as positive control and bovine serum albumin (BSA) as negative control (mean±s.d., *n*=3 experiments). (**e**) Formation of a covalent acetyl-enzyme intermediate monitored by radioactive incorporation. Purified sCASD1-wt and sCASD1-S94A were incubated with [^3^H]acetyl-CoA and the radioactivity incorporated into protein was measured by scintillation counting. BSA was used as negative control (mean±s.d., *n*=4 experiments). (**f**) Verification of the acetyl-enzyme intermediate by mass spectrometry. Purified sCASD1-wt and sCASD1-S94A were incubated with or without acetyl-CoA. After separation by SDS–PAGE and tryptic digestion of the excised protein bands, each sample was analysed by LC-ESI-MS. Theoretical *m*/*z* values are given for the twofold charged tryptic peptides containing the active site serine S94 or the corresponding S94A mutation. Spectra were acquired by accumulating scans within±0.1 min around the respective peak maxima in the extracted ion chromatograms of the given *m*/*z* values (Supplementary Fig. 3). The identity of each peptide variant was confirmed by LC-ESI-MS/MS (Supplementary Fig. 2). Please see Supplementary Figs 9 and 10 for uncropped versions of gel and blot shown in b and of the blot shown in c, respectively.

To analyse the enzymatic activity of CASD1 *in vitro*, we generated a construct that allowed the expression of a soluble secreted form of CASD1 (termed sCASD1), which encompassed the SGNH-like luminal domain and a C-terminal Myc-His_6_-tag (Fig. 2a–c). This form was produced in baculovirus-infected Sf9 insect cells, either as wild-type variant (sCASD1-wt) or as active site serine mutant (sCASD1-S94A). Affinity purified proteins were then used as an enzyme source for *in vitro* assays. First, we analysed whether sCASD1 possesses esterase activity, since the GDSL/SGNH fold is usually found among hydrolytic enzymes^38^, including the sialate 9-*O*-acetylesterase of *Escherichia coli* O157:H7 and the sialate 9-*O*-acetylesterase domains of the haemagglutinin-esterase (HE) proteins of influenza C and bovine coronavirus (BCoV)^41,42,43^. However, under conditions that displayed high *O*-acetyltransferase activity for BCoV-HE, no activity was observed for sCASD1-wt, neither towards the synthetic acetylesterase substrate para-nitrophenylacetate (pNP-acetate; Fig. 2d) nor towards natural *O*-acetylated sialoglycoconjugates (not shown). Thus, while the N-terminal luminal domain of CASD1 displays the characteristic fold of an esterase^39^, it does not appear to function as such. We therefore tested whether sCASD1 might catalyse the reverse reaction and act as a SOAT. Indeed, in the presence of [^3^H]acetyl-CoA, sCASD1-wt showed acetyltransferase activity, resulting in the formation of radiolabelled protein, even in the absence of acceptor substrates (Fig. 2e). No radioactive incorporation was seen in parallel experiments with the mutant sCASD1-S94A, demonstrating that the observed acetyl transfer was enzyme catalysed. SOAT activity of sCASD1 towards its own *N*-glycans was improbable. Although sCASD1 is *N*-glycosylated (Fig. 2c), glycoproteins that are produced in Sf9 cells typically bear pauci mannosidic *N*-glycans lacking penultimate galactose and terminal Sia residues^44^. Consequently, we asked whether sCASD1 can catalyse the formation of a covalent acetyl-enzyme intermediate that could be trapped in the absence of acceptor substrate. Therefore, purified sCASD1 was incubated in the presence or absence of acetyl-CoA, digested with trypsin and analysed by liquid chromatography-electrospray ionization-mass spectrometry (LC-ESI-MS). After incubation of sCASD1-wt with acetyl-CoA, the peptide containing the active site residue S94 (87-HIAFIGDSR-95) occurred as a mixed population of unmodified and modified peptide with a mass shift of 42 Da (shift by *m*/*z*=21 for the doubly charged ion), consistent with the formation of an acetyl adduct (Fig. 2f; note that the sequence of the peptides was confirmed by tandem MS/MS; see Supplementary Fig. 2). Analysis of the peak areas in the extracted ion chromatograms revealed that about 19% of the peptide population was acetylated (Supplementary Fig. 3). In parallel experiments with sCASD1-S94A, only the respective unmodified peptide (87-HIAFIGDAR-95) was found (Fig. 2f; Supplementary Figs 2 and 3), indicating that the hydroxyl group of S94 serves as attachment site for the acetyl group.

### CASD1 catalyses 9-*O-*acetylation of CMP-Neu5Ac

**Figure 3:**
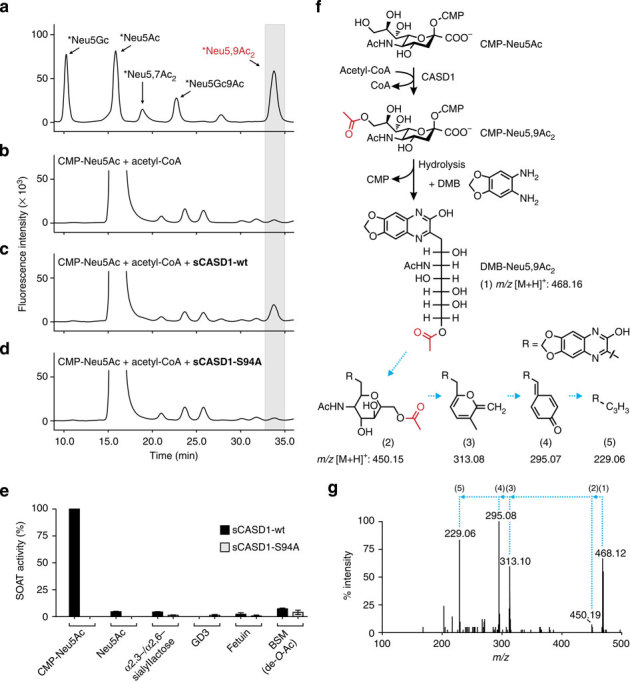
*In vitro* SOAT activity of sCASD1 monitored by DMB-HPLC and LC-ESI-MS. (**a**) HPLC elution profile of the DMB-derivatized Sia reference panel, which included also two non-human Sia species, that is, Neu5Gc (5-*N*-glycolyl neuraminic acid) and 9-*O*-acetylated Neu5Gc (Neu5Gc9Ac). Asterisks denote that the indicated Sia species are labelled with DMB. (**b-d**) HPLC elution profiles of DMB-derivatized samples obtained after incubation of CMP-Neu5Ac and acetyl-CoA without enzyme (**b**), with sCASD1-wt (**c**) or with sCASD1-S94A (**d**). In all profiles, the retention time corresponding to DMB-Neu5,9Ac_2_ of the reference panel is highlighted by a grey box. (**e**) SOAT activity of sCASD1 towards different acceptor substrates. Purified sCASD1-wt and sCASD1-S94A were incubated with acetyl-CoA and the indicated acceptor substrates. Product analysis was performed by DMB-HPLC analysis and the amount of DMB-Neu5,9Ac_2_ was quantified by integrating the corresponding peak areas. Parallel HPLC runs of reactions without CASD1 were used to measure the background, and data are presented as background subtracted data with the values obtained for CMP-Neu5Ac in the presence of sCASD1-wt and acetyl-CoA set to 100% (mean±s.d., *n*=3). (**f**) Scheme showing sCASD1-catalysed 9-*O*-acetylation of CMP-Neu5Ac, subsequent DMB labelling of the reaction product, and the calculated *m*/*z* value ([M+H]^+^) for DMB-derivatized Neu5,9Ac_2_. Structures and calculated *m*/*z* values ([M+H]^+^) of the fragmentation compounds of DMB-Neu5,9Ac_2_ are given according to Klein *et al.*^45^. (**g**) LC-ESI-MS/MS analysis. In the HPLC runs shown in (**b**–**d**), the peak material eluting at the retention time of DMB-Neu5,9Ac_2_ was collected and subjected to LC-ESI-MS/MS. Ions at *m*/*z* 468.16 ([M+H]^+^), indicating DMB-Neu5,9Ac_2_, were obtained only for the material collected from **c** and subsequent fragmentation revealed the three ions (*m*/*z* 313, 295 and 229) that are characteristic for DMB-Neu5,9Ac_2_ (ref. 45). Numbers in brackets above the spectrum refer to the structures given in **f**. DMB, 1,2-diamino-4,5-methylenedioxy-benzene.

To test SOAT activity, purified sCASD1 was incubated with acetyl-CoA and Sia-containing acceptor substrates. After the reaction, Sia was released by acidic hydrolysis to allow derivatization with 1,2-diamino-4,5-methylenedioxy-benzene (DMB) at the free reducing end. The obtained fluorescent Sia derivatives were analysed by reversed-phase high-performance liquid chromatography (HPLC; Fig. 3a-e). SOAT activity was clearly detected towards CMP-Neu5Ac (as indicated by an additional peak in the HPLC profile, which eluted at the same retention time as DMB-derivatized 9-*O-*acetylated Neu5Ac (Neu5,9Ac_2_) of the reference panel; see Fig. 3a,c). The additional peak was not detected in samples obtained from parallel reactions without enzyme (Fig. 3b) or from reactions with sCASD1-S94A (Fig. 3d). Compared with free Neu5Ac or sialoglycoconjugates, CMP-Neu5Ac was by far the best acceptor substrate (Fig. 3e). The identity of the product peak obtained with CMP-Neu5Ac was confirmed by LC-ESI-MS/MS, which showed the expected parent ion mass of DMB-labelled mono-*O*-acetylated Neu5Ac and a fragmentation pattern characteristic for DMB-Neu5,9Ac_2_ (ref. 45; Fig. 3f,g). Together, these data demonstrate that the isolated SGNH-like domain of CASD1 displays SOAT activity *in vitro* and transfers acetyl groups from acetyl-CoA to CMP-Neu5Ac, resulting in the formation of CMP-Neu5,9Ac_2_.

### CASD1 mediates 9-*O-*acetylation of cellular sialoglycans

To study the enzymatic function of CASD1 in a cellular context, we first screened for a mammalian cell line that lacks *O-*acetylated sialoglycans. As a detection tool, we made use of the haemagglutinin-esterase (HE) protein of BCoV. BCoV uses 9-*O*-acetylated sialoglycans as host cell receptors and the HE contains both a lectin domain and a receptor-destroying sialate 9-*O*-acetylesterase domain^42,46^. Inactivation of the esterase activity by S40A mutation (HE^0^) and fusion to the Fc part of human IgG gave rise to a soluble virolectin (BCoV-HE^0^-Fc) that selectively recognizes 9-*O*-acetylated Sias^42^, as confirmed by glycan array screening^47^. Immunofluorescence analysis with BCoV-HE^0^-Fc revealed that several mammalian cell lines express 9-*O*-acetylated sialoglycotopes exclusively in the Golgi apparatus (Supplementary Fig. 4a). In Chinese hamster ovary (CHO) cells, the underlying carrier structures were *N*- and *O*-glycans, since blocking *O*-glycosylation sufficiently abrogated 9-*O*-acetylation only in cells that additionally lacked complex and hybrid *N*-glycans (Supplementary Fig. 5).

**Figure 4:**
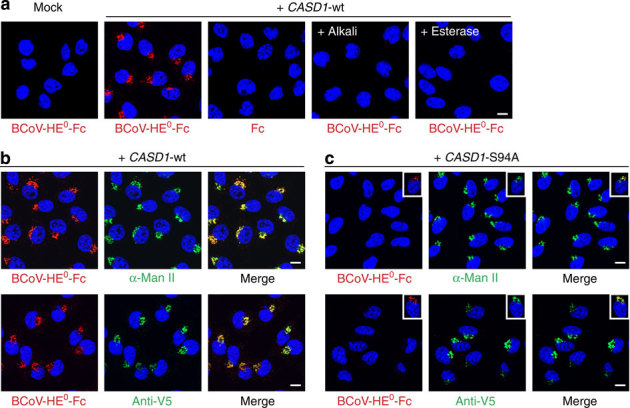
CASD1 mediates 9-*O-*acetylation of cellular sialoglycoconjugates. (**a**) LM-*TK*^−^ cells were transfected with empty vector (mock) or a plasmid encoding epitope-tagged CASD1 (+*CASD1*-wt). After fixation and permeabilization with Triton X-100, cells were stained with the virolectin BCoV-HE^0^-Fc, which recognizes 9-*O*-acetylated Sia. The specificity of the virolectin stain was controlled using human Fc fragments instead of virolectin (Fc) or by de-*O*-acetylation before the virolectin staining. De-*O*-acetylation was performed by alkali treatment (+alkali) or incubation with BCoV-HE-Fc harbouring an enzymatically active esterase domain (+esterase). Nuclei were counterstained with DAPI (blue). (**b**,**c**) LM-*TK*^−^ cells stably expressing V5-tagged wild-type CASD1 (*+CASD1*-*wt*) or CASD1 carrying the mutation S94A (*+CASD1-S94A*) were co-stained with BCoV-HE^0^-Fc (red) and anti-α-Man II pAb (green) or BCoV-HE^0^-Fc (red) and anti-V5 mAb (green) as shown in the upper and lower panel, respectively. Nuclei were stained with DAPI (blue). Insets in **c** show a representative cell of a minor cell population (<1%), which stained positively with BCoV-HE^0^-Fc. Scale bar, 10 μm. DAPI, 4,6-diamidino-2-phenylindole.

Importantly, all cell lines that were positive for 9-*O*-acetylation were also positive for *CASD1* transcripts (Supplementary Fig. 4b). In fact, we found only a single cell line, the murine fibroblast cell line LM-*TK*^−^, that was devoid of both (Supplementary Fig. 4). Transfection of this naturally occurring *O*-Ac-negative cell line with *CASD1* complementary DNA (cDNA), but not empty vector, led to the formation of Golgi-resident 9-*O*-acetylated sialoglycotopes as shown by virolectin staining (Fig. 4a). De-*O*-acetylation, by either alkali treatment or the sialate 9-*O*-acetylesterase activity of BCoV-HE, completely abolished the virolectin signals and was used as specificity control (Fig. 4a). After stable expression of wild-type CASD1, 9-*O*-acetylated sialoglycotopes were detected in all cells and co-localized with the Golgi marker α-Man II and Golgi-localized V5-tagged CASD1 (Fig. 4b). Introduction of the S94A exchange into full-length CASD1 (CASD1-S94A) resulted in a loss of function. Although the mutant protein was expressed and localized to the Golgi, virolectin staining was seen only in <1% of the cells (Fig. 4c). Taken together, these experiments demonstrate that CASD1 mediates sialate 9-*O*-acetylation in LM-*TK*^−^ cells and that this function is impaired by alanine exchange of S94.

### *CASD1* knockout abolishes Sia *O-*acetylation in HAP1 cells

**Figure 5:**
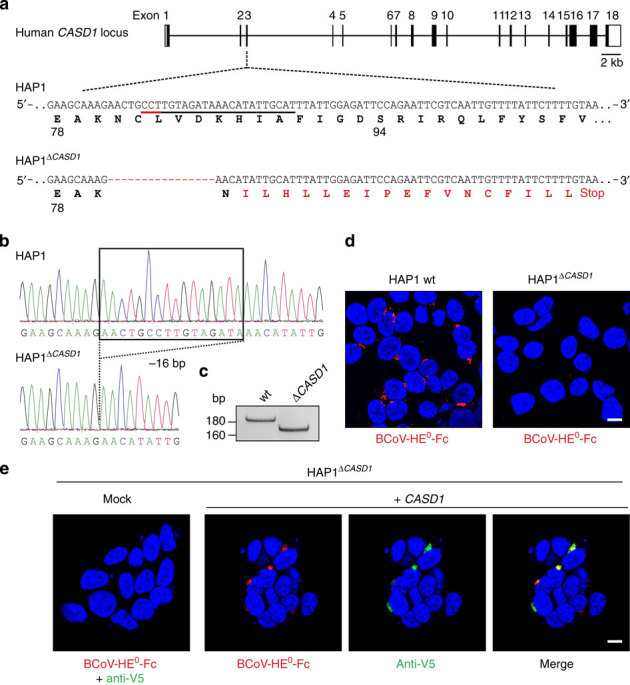
*CASD1* knockout results in a loss of Sia 9-*O*-acetylation in HAP1 cells. (**a**) Schematic representation of the human *CASD1* locus showing the target site used for CRISPR/Cas-mediated genome editing in HAP1 cells. Target sequence and protospacer adjacent motif are underlined (black and red line, respectively). A 16-bp frame shift mutation found in the knockout cell line (HAP1^Δ*CASD1*^) is depicted in red together with the resulting changes in the translation product. (**b**) Sequence analysis of the target site. Exon 3 was amplified from genomic DNA and analysed by Sanger sequencing. (**c**) PCR with reverse transcription analysis demonstrating the presence of a microdeletion in *CASD1* transcripts of HAP1^Δ*CASD1*^ cells. (**d**) Immunofluorescence analysis of Triton-permeabilized HAP1 wild-type (wt) and HAP1^Δ*CASD1*^ cells stained with BCoV-HE^0^-Fc. Nuclei were counterstained with DAPI (blue). (**e**) Complementation of HAP1^Δ*CASD1*^ with *CASD1* cDNA. HAP1^Δ*CASD1*^ cells were transiently transfected with empty vector (mock) or a plasmid encoding epitope-tagged CASD1 (+*CASD1*). Triton-permeabilized cells were co-stained with BCoV-HE^0^-Fc (red) and anti-V5 mAb (green). Nuclei were counterstained with DAPI (blue). Scale bar, 10 μm. DAPI, 4,6-diamidino-2-phenylindole.

To confirm that CASD1 plays a crucial role in the formation of 9-*O*-acetylated sialoglycans, we generated a *CASD1* knockout in the near-haploid human cell line HAP1 using the CRISPR/Cas9 (clustered regularly interspaced short palindromic repeats/CRISPR-associated 9) system^48^. The obtained knockout cell line (HAP1^Δ*CASD1*^) harbours a 16-bp frame shift mutation in exon 3 of *CASD1*, allowing only the production of a truncated polypeptide that already lacks S94, the most N-terminally located residue of the catalytic triad (Fig. 5a-c). Whereas parental HAP1 cells expressed 9-*O*-acetylated sialoglycans in the Golgi apparatus, 9-*O*-acetylation was completely lost in HAP1^Δ*CASD1*^ cells (Fig. 5d). This was further confirmed by DMB-HPLC analysis revealing the presence of DMB-derivatized Neu5,9Ac_2_ in parental but not in HAP1^Δ*CASD1*^ cells (Supplementary Fig. 6). Transfection with *CASD1* cDNA, but not empty vector, resulted in successful complementation of the loss-of-function defect and restored 9-*O*-acetylation of Golgi-localized sialoglycotopes (Fig. 5e). Of note, a genetic knockout carried out in parallel in human embryonic kidney (HEK) 293T cells showed the same outcome (Supplementary Fig. 7) and thus provided independent evidence for the essential role of CASD1 in Sia 9-*O*-acetylation.

### CASD1 mediates 9-*O*-acetylation of gangliosides

**Figure 6:**
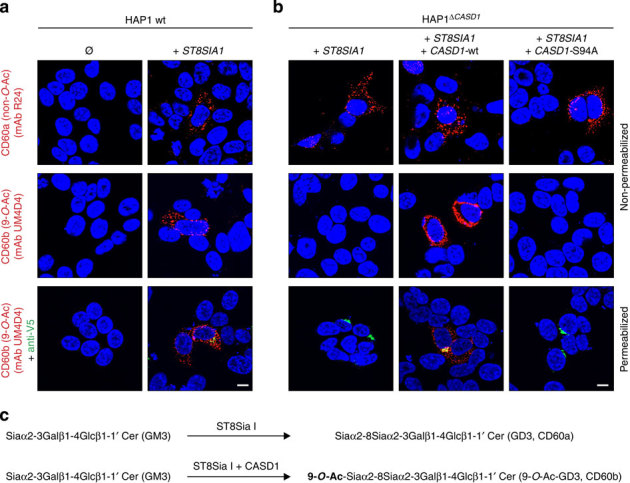
CASD1 mediates 9-*O*-acetylation of gangliosides. (**a**) Parental, wild-type HAP1 cells (HAP1 wt) were analysed for the expression of GD3 and 9-*O*-Ac-GD3 before (Ø) and after transient expression of V5-tagged ST8Sia I (+*ST8SIA1*). Non-permeabilized cells were stained with anti-CD60a mAb R24 (upper panel) or anti-CD60b mAb UM4D4 (middle panel). Triton-permeabilized cells (lower panel) were co-stained with mAb UM4D4 (red) and anti-V5 mAb (green). Nuclei were counterstained with DAPI (blue). (**b**) HAP1^Δ*CASD1*^ cells were transiently transfected with *ST8SIA1* cDNA either alone (+*ST8SIA1*), together with wild-type *CASD1* cDNA (+*ST8SIA1*+*CASD1*-wt) or together with mutated *CASD1* cDNA (+*ST8SIA1*+*CASD1*-S94A). Non-permeabilized cells were stained with mAb R24 (upper panel) and mAb UM4D4 (middle panel) and Triton-permeabilized cells (lower panel) were co-stained with mAb UM4D4 (red) and anti-V5 mAb (green). Nuclei were counterstained with DAPI (blue). Scale bar, 10 μm. (**c**) Scheme showing the conversion of GM3 to GD3 and the formation of 9-*O*-Ac-GD3. DAPI, 4,6-diamidino-2-phenylindole.

On the basis of HAP1 and HAP1^Δ*CASD1*^ cells, we next explored the role of CASD1 in the 9-*O*-acetylation of gangliosides such as GD3. To do so, we made use of a set of monoclonal antibodies (mAbs) that distinguish between GD3 (CD60a) and 9-*O*-acetylated GD3 (CD60b). HAP1 cells lack GD3 but can generate the disialo ganglioside after expression of the sialyltransferase ST8Sia I (Fig. 6a; mAb R24). ST8Sia I converts GM3 to GD3 by adding an α2,8-linked Sia residue, which in turn can carry an *O*-acetyl group in C9 position (Fig. 6c)^49^. In HAP1 wild-type cells, expression of ST8Sia I resulted in the formation of both CD60a and CD60b antigens, as shown by additional staining with mAb UM4D4 (Fig. 6a). As described for other anti-CD60b antibodies, UM4D4 recognizes 9-*O*-acetylated GD3 and similar gangliosides possessing a terminal 9-*O*-acetylated disialo group such as 9-*O*-acetylated disialo neolactotetraosylceramide and 9-*O*-acetylated disialo neolactohexaosylceramide (see Supplementary Fig. 8c for details on ganglioside structures)^50^. However, binding of mAb UM4D4 strictly relies on the *O*-acetylation of the carbohydrate epitope and no cross-reactivity towards the unmodified epitope is seen (see refs 50, 51 and Supplementary Fig. 8b). Newly synthesized CD60a and CD60b epitopes were both detected at the cell surface, as demonstrated by respective signals on non-permeabilized cells. In HAP1^Δ*CASD1*^ cells, expression of ST8Sia I alone led to the formation of GD3 (Fig. 6b; mAb R24) but not 9-*O*-Ac-GD3 or other 9-*O*-acetylated gangliosides of the neolacto series (Fig. 6b; mAb UM4D4). Ganglioside modification was only detected after co-expression of ST8Sia I with CASD1, demonstrating that CASD1 is crucial for the formation of 9-*O*-acetylated CD60b epitopes (Fig. 6b; middle images). Again, alanine exchange of the active site residue S94 drastically impaired CASD1 function (Fig. 6b; right images).

The critical role of CASD1 for the formation of 9-*O*-acetylated CD60b epitopes was further confirmed in HEK293T wt and HEK293T^Δ*CASD1*^ cells by immunofluorescence and thin-layer chromatography (TLC) analysis, whereas no indication for the presence of 7-*O*-acetylated disialo gangliosides was found (Supplementary Fig. 8a,b).

## Discussion

Here we define CASD1 as the key factor for Sia 9-*O*-acetylation, the most common Sia modification in humans. Pivotal to our study was the observation that mammalian cell lines frequently express 9-*O*-acetylated sialoglycans in the Golgi apparatus, even if 9-*O*-acetylation of cell surface glycans is non-detectable. This phenomenon, described only by one previous study^37^, highlights that 9-*O*-acetylation is much more common than anticipated from cell surface staining. The finding that Golgi-confined 9-*O*-acetylated Sias were attached to *N*- and *O*-glycans suggests Golgi-resident glycoproteins as underlying carriers and interestingly, 9-*O*-acetylated Sia was recently found on the *N*-glycans of a human sialyltransferase^52^.

As shown for HAP1 and HEK293T cells, CASD1 is essential and sufficient for Golgi-confined 9-*O*-acetylation. However, 9-*O*-acetylation of cell surface molecules such as GD3 and similar disialo gangliosides required the additional expression of ST8Sia I. This indicates that parental HAP1 cells lack detectable cell surface 9-*O*-acetylation, because cell surface sialoglycotopes amenable to 9-*O*-acetylation are either absent or present in very low quantities. Regulation of cell surface 9-*O*-acetylation by the action of specific sialyltransferases is consistent with data from CHO cells, showing that cell surface 9-*O*-acetylation could be induced by ST8Sia I or ST6Gal I, two sialyltransferases that are normally not expressed in CHO cells^53^.

Our *in vitro* data establish that sCASD1, a soluble CASD1 variant encompassing only the SGNH-like domain, transfers acetyl groups from acetyl-CoA to CMP-Neu5Ac, the donor substrate of all sialyltransferases. Although the situation may be more complex *in vivo*, this result suggests that CASD1 may not directly 9-*O*-acetylate sialoglycoconjugates, but instead provides 9-*O*-acetylated CMP-Neu5Ac (CMP-Neu5,9Ac_2_) for sialyltransferases. In this scenario, the sialyltransferases would play a critical role in the biosynthesis of glycoconjugates that carry 9-*O*-acetylated Sia. In support of this, CMP-Neu5,9Ac_2_ was detected in bovine submandibular glands^54^ and biochemical data obtained with human colon mucosa showed a Golgi-associated SOAT activity that utilized CMP-Neu5Ac as acceptor and a sialyltransferase activity that transferred 9-*O*-acetylated Sia from CMP-Neu5,9Ac_2_ to endogenous and exogenous acceptors^33,55^. Moreover, the use of CMP-Neu5,9Ac_2_ has been shown for several sialyltransferases and is exploited for chemo-enzymatic synthesis of 9-*O*-acetylated sialosides^56^. *In vivo*, the efficient and selective usage of CMP-Neu5,9Ac_2_ may rely on a physical interaction between CASD1 and specific sialyltransferases.

Although our data demonstrates that CASD1 mediates 9-*O*-acetylation, we cannot rule out the possibility that the enzyme initially transfers the acetyl group to the C7 position of Sia followed by migration to position C9 in a non-enzymatic step. Even under physiological conditions, *O*-acetyl groups at the Sia side chain can spontaneously migrate from C7 and C8 to an unsubstituted hydroxyl group at position C9, where the *O*-acetyl group is more stable^57,58^. Moreover, 7-*O*-Ac-specific SOAT activity has been detected in several tissues^31,59,60^ and studies on microsomal fractions from bovine submandibular glands provided evidence for an enzyme that accelerates the migration of *O*-acetyl groups from C7 to C9 *in vivo*^59^. In line with previous studies on 7-*O*-Ac-specific SOAT activity, Arming *et al.*^35^ observed a role of CASD1 in 7-*O*-acetylation but not in 9-*O*-acetylation of GD3 as shown by FACS analysis with anti-CD60c and anti-CD60b mAbs^35^. The stability of *O*-acetyl groups at C7 might thus be cell-type-specific and could explain the discrepancy with our data.

By identification of a covalent acetyl-enzyme intermediate that involves the active site residue S94, we provide first insight into the catalytic mechanism of CASD1. Transfer of an acetyl group from acetyl-CoA to a serine placed in a Ser–His–Asp catalytic triad is reminiscent of what we found for the polysialic acid-specific *O*-acetyltransferase OatC of *Neisseria meningitidis*^61^ and suggests a ping–pong mechanism as described for other members of the SGNH-hydrolase family^62,63^. Consistently, we propose a mechanism in which the catalytic triad of CASD1 forms a charge relay network to activate S94, allowing nucleophilic attack of the carbonyl carbon of the acetyl donor. This leads to the transfer of the acetyl group to S94, followed by transfer to CMP-Neu5Ac during the second half reaction. Alanine exchange of S94 in sCASD1 prevented the formation of the acetyl-enzyme intermediate and resulted in a complete loss of SOAT activity *in vitro*.

The soluble sCASD1 variant used for *in vitro* experiments encompassed only the SGNH-like domain, whereas *in vivo*, this domain is fused to a C-terminal multi-TM domain. Detection of residual virolectin staining in cells expressing full-length CASD1-S94A may indicate that the C-terminal part contributes to CASD1 activity *in vivo*. First indication for this scenario is given by a bioinformatics analysis of the cryptococcal Cas1 protein (Cas1p)^39^. This study highlighted that the multi-TM domain found in Cas1p and its animal orthologue CASD1 shares homology with bacterial multi-TM *O*-acetyltransferases that are involved in the modification of cell surface or periplasmic biopolymers such as polysaccharides and peptidoglycan^39^. Of note, several of the bacterial multi-TM proteins work in concert with an SGNH-like *O*-acetyltransferase, with the latter being expressed either as separate protein or fused to the multi-TM part as in CASD1 (refs 62, 64, 65).

In conclusion, we characterized human CASD1 as key enzyme for Sia 9-*O*-acetylation and provided first insight into the catalytic mechanism. This opens new experimental avenues for the development of effective therapeutic strategies targeting CASD1 to regulate pathological changes in the 9-*O*-acetylation status and to combat drug-resistant cancer cells in ALL, whose survival crucially depend on 9-*O*-acetylation^23^.

## Methods

### Homology modelling

The three-dimensional structure of the catalytic domain of CASD1 was modelled using the Phyre2 protein fold recognition server^66^. On the basis of the structure of the isoamyl acetate-hydrolysing esterase from *Saccharomyces cerevisiae* (PDB accession code 3mil), which was identified as top rank template, residues 83-290 of human CASD1 were modelled with 99.3% confidence.

### Generation of mammalian expression plasmids

To generate a construct encoding full-length CASD1 with an N-terminal V5 and a C-terminal Myc epitope (V5-CASD1-Myc), the coding region of human *CASD1* (accession no. NM_022900) was amplified by PCR using the primers 5′-GCTCGGGATCCGCGGCTCTGGCCTACAACCTG-3′ and 5′-GCTCGCTCGAGATGTTTTGATTTATCTTGAATGGATG-3′ containing BamHI and XhoI restriction sites (underlined), respectively, and the resulting PCR product was ligated into the corresponding restriction sites of the vector pcDNA3 (Invitrogen). Sequences encoding the epitope tags were inserted by adapter ligation. For the V5 epitope, the pre-hybridized oligonucleotide pair 5′-AGCTTCGAATGGGTAAGCCTATCCCTAACCCTCTCCTCGGTCTCGATTCTACGG-3′ and 5′-GATCCCGTAGAATCGAGACCGAGGAGAGGGTTAGGGATAGGCTTACCCATTCGA-3′ was ligated into the HindIII and BamHI sites of pcDNA3. For the Myc epitope, the pre-hybridized oligonucleotide pair 5′-TCGAGGAACAAAAACTCATCTCAGAAGAGGATCTGAATTAAT-3′ and 5′-CTAGATTAATTCAGATCCTCTTCTGAGATGAGTTTTTGTTCC-3′ was ligated into the XhoI and XbaI sites of pcDNA3, resulting in the plasmid pcDNA3-V5-CASD1(wt)-Myc.

Site-directed mutagenesis was performed by PCR using the QuikChange site-directed mutagenesis kit (Stratagene) and pcDNA3-V5-CASD1(wt)-Myc as template. To introduce the amino-acid exchange S94A, the mutagenesis primers 5′-GCATTTATTGGAGATGCCAGAATTCGTCAATTG-3′ and 5′-CAATTGACGAATTCTGGCATCTCCAATAAATGC-3′ were used, resulting in the plasmid pcDNA-V5-CASD1(S94A)-Myc.

For expression of the human sialyltransferase ST8Sia I, a full-length construct encoding an N-terminal V5 epitope was generated by amplification of the coding region of human *ST8SIA1* (I.M.A.G.E. clone IRCMp5012B0613D, ImaGenes) using the primers 5′-GCTAAGCTTCGA**ATGGGTAAGCCTATCCCTAACCCTCTCCTCGGTCTCGATTCTACG**GGTACCAGCCCCTGCGGGCGGGC-3′ and 5′-GCTGCGGCCGCCTAGGAAGTGGGCTGGAGTG-3′ containing the sequence encoding the V5 epitope (bold), HindIII and NotI restriction sites (underlined). The resulting PCR fragment was ligated into the HindIII and NotI sites of the expression vector pcDNA3.1-zeo (Invitrogen).

To generate constructs encoding a soluble Fc-chimera of BCoV-HE, DNA encoding the ectodomain (residues 1-388) of BCoV-HE (accession no. AAL57307) was generated by gene synthesis (Eurofins MWG Operon) and subsequent amplification by PCR using the primers 5′-GACTGCTAGCATGTTTTTGCTTCCTAGATTTG-3′ and 5′-GACTGGATCCACTTACCTGTATCATACACACAAATAGGTAC-3′ containing NheI and BamHI restriction sites (underlined), respectively. The obtained PCR product was ligated into the NheI and BamHI sites of pcDNA3.1-zeo, upstream of the sequence encoding the Fc part of human IgG1. A corresponding plasmid that encodes BCoV-HE^0^-Fc harbouring an inactive esterase domain due to the amino-acid exchange S40A was generated by site-directed mutagenesis using the QuikChange site-directed mutagenesis kit (Stratagene) and the mutagenesis primers 5′-GGAGATTGGTTTTTATTTGGTGACGCCCGTTCAGATTG-3′ and 5′-CAATCTGAACGGGCGTCACCAAATAAAAACCAATCTCC-3′. The identity of all constructs was confirmed by sequencing.

### Mammalian cell lines and growth conditions

The murine fibroblast cell line LM-*TK*^−^ (ATCC CCL1.3), the human rhabdomyosarcoma cell line TE671, HEK293T cells and CHO wild type (CHO-K1), CHO lec1, and CHO 6B2 cells were maintained in DMEM/Ham’s F-12 1:1 (Biochrom) supplemented with 1 mM sodium pyruvate and 10% fetal calf serum (FCS) at 37 °C and 5% CO_2_. Madin Darby canine kidney I cells (MDCK I) were cultured in DMEM (containing 450 mg l^−1^ glucose; Biochrom) supplemented with 10% FCS at 37 °C and 5% CO_2_. Additional information on the used cell lines are given in the legends to Supplementary Figs 4 and 5. The near-haploid human cell line HAP1 was purchased from Haplogen Genomics (Austria) and maintained in Iscove’s modified Dulbecco’s medium (Gibco) supplemented with 5% FCS at 37 °C and 5% CO_2_. Hybridoma cells producing mAb R24 (ATCC HB-8445) were maintained in RPMI 1640 (Sigma) adjusted to contain 4.5 g l^−1^ glucose, 10 mM Hepes, 1 mM sodium pyruvate and 10% FCS. All cell lines were routinely screened for mycoplasma contamination using the PlasmoTest detection kit (InvivoGen).

Inhibition of mucin-type *O*-glycosylation was achieved by cultivating CHO wild-type and CHO lec1 cells for 3 weeks in medium supplemented with 2 mM benzyl-*N*-acetyl-α-galactosaminide (benzyl-α-GalNAc; Calbiochem).

### Transfection of mammalian cells

Transfection of LM-*TK*^−^ and CHO cells was performed with Lipofectamine (Invitrogen). Briefly, cells were grown in six-well plates (70-80% confluency), washed twice with PBS and transfected with 1 μg of plasmid DNA and 6 μl Lipofectamine in 1 ml of OptiMEM (Gibco). After 7 h at 37 °C and 5% CO_2_ the transfection was stopped by adding 1 ml of culture medium supplemented with 10% FCS. For HAP1 and HEK293T cells, the protocol was varied by using 2 μl TurboFectin 8.0 (OriGene Technologies) or 2 μl polyethylenimine ‘Max’ (Polyscience) instead of Lipofectamine. For the selection of stable transfectants, 750 μg ml^−1^ G418 (Calbiochem) or 750 μg ml^−1^ zeocin (Life Technologies) were added 72 h after start of the transfection. Colonies were picked and cloned by limiting dilution.

### CRISPR/Cas-mediated genome editing

*CASD1* knockout cells were generated by Haplogen Genomics (Austria) based on the near-haploid human cell line HAP1 and CRISPR/Cas-mediated genome editing^48^. In brief, HAP1 cells were edited using the *Streptococcus pyogenes* nuclease Cas9, together with a guide RNA containing a gene-specific sequence targeting exon 3 of *CASD1* (5′-ATGCAATATGTTTATCTACA-3′). *CASD1* knockout HEK293T cells were generated by CRISPR/CAS-mediated genome editing essentially as described by Zheng *et al.*^67^ with two guide RNAs targeting residues −33 to −14 and 38 to 57 of *CASD1* exon 1 (5′-GTCCGCCGCGCACTGTTGTCA-3′ and 5′-GTCAACCACTACTTCAGCGTG-3′, respectively). Single cell clones of HAP1 and HEK293T cells were isolated by limiting dilution and genotyped by PCR and DNA sequencing using the primers 5′-GGAGTGGTAAGTTAAACAAACCTGG-3′ and 5′-TTTTTCAGCTCCTACTCAATCTGGA-3′ (for HAP1 cells) and 5′-GCTATTAAGGCAGGAGGCTAAAG-3′ and 5′-AACCACCAGCCATTGTCCTTTG-3′ (for HEK293T cells).

### Reverse transcription–PCR analysis

RNA was prepared from mammalian cell lines using TRIzol reagent (Invitrogen) according to the manufacturer’s instructions. cDNA was generated from 1 μg of RNA using Revert Aid premium reverse transcriptase (Fermentas) and random hexamer primers according to the manufacturer’s recommendations. Gene-specific cDNA was amplified using the following primer pairs: 5′-GTTTGGCAACCTCACAGTTGTATGATG-3′ and 5′-AGTTTCTCTGCTCGATTCAG-3′ (*CASD1*), and 5′-CACACCTTCTACAATGAGCTG-3′ and 5′-GTCAGGTCCCGGCCAGCCAG-3′ (*ß*-*ACTIN*). PCR products were separated on a 2.5% agarose gel and stained with ethidium bromide. To confirm the presence of a 16-bp microdeletion in *CASD1* transcripts of HAP1^Δ*CASD1*^ cells, *CASD1*-specific cDNA was amplified using the primer pair 5′-CGCCTCCCGCCGCTACCGAGG-3′ and 5′-CAATTGACGAATTCTGGAATCTC-3, which allows the amplification of a 184- and 168-bp PCR product from wild-type and mutated *CASD1*, respectively. The obtained PCR products were separated on a 6% polyacrylamide gel and stained with ethidium bromide.

### Production and purification of BCoV-HE-Fc and BCoV-HE^0^-Fc

BCoV-HE^0^-Fc and BCoV-HE-Fc were produced by transient expression in HEK293T cells and purified from the tissue culture supernatant as described^42^. As an alternative source of recombinant proteins, CHO cells stably transfected with plasmids encoding BCoV-HE-Fc or BCoV-HE^0^-Fc were cultivated in DMEM/Ham’s F-12 1:1 (Biochrom) containing 1 mM sodium pyruvate and 5% low IgG FCS (Gibco). Tissue culture supernatants from transfected HEK293T cells and conditioned medium of the CHO cells were collected and adsorbed to Protein A Sepharose (GE Healthcare). After washing with 20 column volumes of 20 mM sodium phosphate pH 7.0, bound protein was eluted with 0.1 M glycine-HCl pH 2.8 and neutralized by 1 M Tris-HCl pH 8.5. After addition of NaCl to a final concentration of 150 mM, purified Fc chimeras were stored at -80 °C.

### Expression and purification of sCASD1

Plasmids enabling the secreted expression of sCASD1 in insect cells were based on a modified pFastBac1 vector (Invitrogen) carrying sequence stretches encoding an N-terminal honeybee melittin signal peptide (HBM) and a C-terminal Myc-His_6_-tag. The sequence encoding residues 39–304 of human CASD1 was amplified by PCR using the primers 5′-GCTCGGAGCTCCGCCTCCCGCCGCTACCGAGG-3′ and 5′-GCTCGTCTAGATTGACAACAGGACCCATCTACAGG-3′, containing SacI and XbaI restriction sites (underlined), and either pcDNA-V5-CASD1(wt)-Myc or pcDNA-V5-CASD1(S94A)-Myc as template. The resulting PCR products were ligated into the restriction sites of the modified pFastBac1 vector, resulting in the plasmids pFastBac1-HBM-sCASD1(wt)-Myc-His_6_ and pFastBac1-HBM-sCASD1(S94A)-Myc-His_6_.

*Spodoptera frugiperda* (Sf9) cells (Gibco) were grown at 27 °C in shaking cultures at 80 r.p.m. in protein-free Insect-Xpress medium (Lonza) and maintained at a density of 0.5 × 10^6^ to 6 × 10^6^ viable cells per ml. Transient expression of sCASD1-wt and sCASD1-S94A was performed on infection with recombinant baculovirus particles generated by the Bac-to-Bac system (Invitrogen) and the pFastBac1-based plasmids described above. Conditioned medium was collected 96 h after infection, concentrated 10-fold by tangential flow ultrafiltration (Ultrasette, cutoff of 10 kDa, Pall Corporation), diafiltered against 50 mM sodium phosphate buffer pH 7.5 containing 100 mM NaCl and passed over a HisTrap HP column (GE Healthcare). After washing with 20 column volumes of 50 mM sodium phosphate pH 7.5 containing 100 mM NaCl and 10% glycerol, bound protein was eluted by an imidazole gradient. The imidazole was subsequently removed by gel filtration using a Superdex 200 10/300 GL column (GE Healthcare) equilibrated with 20 mM MES pH 6.8 containing 100 mM NaCl.

### Enzymatic removal of *N*-glycans

To remove *N*-glycans, 2 μg of sCASD1-wt were incubated with two units of PNGaseF (Alexis Biochemicals) in a total volume of 30 μl of 10 mM Tris-HCl pH 8.0, 1 mM EDTA. Digests were performed at 37 °C for the indicated time points.

### SDS–PAGE and immunoblotting

Proteins were separated by SDS–polyacrylamide gel electrophoresis under reducing conditions and proteins were either stained with Coomassie Brilliant Blue (Roth) or transferred to polyvinylidene difluoride membranes (GE Healthcare) for western blot analysis. Myc-tagged proteins were detected using mAb 9E10 (5 μg ml^−1^, Roche), followed by goat anti-mouse IgG horseradish peroxidase (HRP) conjugate (1:20,000; Jackson Immunoresearch) and enhanced chemiluminescence detection.

### Immunofluorescence analysis

Cells were grown on glass coverslips, fixed in 4% paraformaldehyde in PBS for 15 min and washed with PBS. Membrane permeabilization was performed with 0.2% (v/v) Triton X-100 in PBS for 10 min at 4 °C. Selective permeabilization of the plasma membrane by low concentrations of digitonin was performed as described^68^. Antibodies and virolectin were diluted in 1% bovine serum albumin (BSA) in PBS and staining was performed for 1 h at room temperature (RT) using the following concentrations: BCoV-HE^0^-Fc (2.5 μg ml^−1^), anti-Myc mAb 9E10 (15 μg ml^−1^; Roche cat. 11667149001), anti-V5 mAb (1 μg ml^−1^; Acris cat. SM1691PS), rabbit anti-CASD1 pAb 500 (1:300; rabbit serum obtained from immunizations with sCASD1-wt carried out at Aldevron, Freiburg), anti-9-*O*-Ac-GD3 mAb UM4D4 (1 μg ml^−1^; Santa Cruz cat. Sc-32269), anti-GD3 mAb R24 (2.5 μg ml^−1^; purified from cell culture supernatant of hybridoma cells by Protein A affinity chromatography), anti-α-Man II pAb (1:10,000; kindly provided by Kelley Moremen), anti-Giantin pAb (1:1,000; Covance cat. PRB-114C) and human IgG Fc fragments (75 μg ml^−1^; Jackson Immunoresearch). After washing with PBS, cells were incubated for 1 h with one of the following secondary antibodies used in a 1:500 dilution: sheep anti-mouse IgG Cy3-conjugate (Sigma Aldrich cat. C 2181); sheep anti-rabbit IgG Cy3-conjugate (Sigma Aldrich cat. C2306); donkey anti-rabbit IgG Alexa Fluor 488-conjugate (Molecular Probes cat. A21206); donkey anti-mouse IgG Alexa Fluor 488-conjugate (Molecular Probes cat. A 21202); donkey anti-human IgG DyLight500-conjugate (Thermo Scientific cat. SA5-10127); goat anti-mouse IgM Alexa Fluor 568-conjugate (Invitrogen cat. A 21043); or rabbit anti-mouse IgG3 DyLight549-conjugate (Rockland cat. 610-442-043). After washes with PBS, cells were mounted in Vectashield mounting medium containing 4,6-diamidino-2-phenylindole (Vector) and analysed under a Zeiss Axiovert 200 M microscope equipped with ApoTome module, AxioCam MRm digital camera and Axio Vision software (Zeiss).

### De-*O*-acetylation of sialoglycoconjugates

To de-*O*-acetylate sialoglycoconjugates before the analysis with virolectin, fixed and permeabilized cells were incubated with either 0.1 M NaOH or 45 μg ml^−1^ BCoV-HE-Fc in 1% BSA/PBS for 30 min at 37 °C. De-*O*-acetylation of bovine submandibular gland mucin (BSM; Sigma Aldrich) used for *in vitro* assays was performed overnight in 0.1 M triethylamine at 37 °C followed by lyophilisation and resuspension in 50 mM phosphate buffer pH 6.5.

### *In vitro* esterase activity assay

Esterase activity was measured in a total volume of 100 μl containing 15 pmol protein (BCoV-HE-Fc, sCASD1-wt or BSA), 1 mM pNP-acetate (Sigma Aldrich), 50 mM sodium phosphate pH 6.5 and 50 mM KCl. Samples were incubated for 30 min at RT and the absorbance of released pNP was measured at 405 nm.

### Radioactive incorporation assay

For radioactive incorporation assays, 5 μg of purified protein (sCASD1-wt or sCASD1-S94A) was incubated for 15 min at 37 °C in a total volume of 50 μl containing 50 mM phosphate buffer pH 6.5, 50 mM KCl and 2.5 μM [^3^H]acetyl-CoA (0.37 kBq μl^−1^, American Radiolabeled Chemicals). To stabilize the acetyl-enzyme intermediate, reaction mixtures were kept at -20 °C for at least 18 h. After thawing at RT, an aliquot of 10 μl was spotted on Whatman grade 3MM chromatography paper and free radioactivity was removed by descending paper chromatography using 300 mM ammonium acetate pH 7.5/70% ethanol as mobile phase. Radioactivity incorporated into protein remained at the origin and was quantified by scintillation counting.

### *In vitro* formation of the acetyl-enzyme intermediate

Purified sCASD1-wt or sCASD1-S94A (5 μg) was incubated for 15 min at 37 °C with or without 2.5 μM acetyl-CoA (Sigma Aldrich) in 50 μl containing 50 mM phosphate buffer pH 6.5 and 50 mM KCl. After storage at -20 °C for at least 18 h to stabilize the acetyl-enzyme intermediate, the reaction mixture was separated by SDS–PAGE and the protein band was excised, digested with trypsin and analysed by LC-ESI-MS.

### In-gel digestion of proteins with trypsin

On separation by SDS–PAGE and staining with Coomassie Blue (Roth), protein bands were excised from gels and subjected to tryptic digest. Briefly, the gel pieces were dehydrated with acetonitrile and rehydrated with 100 mM NH_4_HCO_3_ containing 10 mM dithiothreitol (Roth). Subsequently, gel pieces were treated with 100 mM NH_4_HCO_3_ containing 100 mM iodoacetamide (Sigma). After a second dehydration step with acetonitrile and rehydration with 100 mM NH_4_HCO_3_ buffer followed by dehydration with acetonitrile, the dried gel pieces were rehydrated with 20 ng μl^−1^ trypsin (Promega) in 50 mM NH_4_HCO_3_ buffer and incubated overnight at 37 °C. Peptides were extracted with 75% acetonitrile containing 0.1% formic acid and dried in a vacuum centrifuge.

### *In vitro* SOAT activity assay using DMB-HPLC analysis

Enzyme assays were performed for 3 h at 37 °C in 20 μl containing 50 mM MES pH 6.5, 10 mM MnCl_2_, 1 mM acetyl-CoA with or without 5 μg sCASD1-wt or sCASD1-S94A. As acceptor substrates, the following compounds were tested: CMP-Neu5Ac (600 μM; Nacalai Tesque); Neu5Ac (600 μM; Nacalai Tesque); a mixture of α2,3- and α2,6-linked sialyllactose (600 μM; Sigma Aldrich); GD3 (600 μM; Calbiochem); fetuin (7 μg; Sigma Aldrich); and de-*O*-acetylated BSM (7 μg). After the enzyme reaction, Sias were released by acidic hydrolysis. To reduce *O*-acetyl group loss and migration during this step, we followed the protocol of Mawhinney and Chance^69^ using 2 M propionic acid for 1 h at 80 °C. Liberated Sias were subsequently derivatized with DMB according to Hara *et al.*^70^. Briefly, Sias were labelled for 2.5 h at 50 °C using 8 mM DMB (Dojindo) in 1.5 M propionic acid containing 0.8 M ß-mercaptoethanol and 14.2 mM sodium hydrosulfite. Fluorescently labelled Sias were separated using the Shimadzu Prominence CBM-20A UFLC system equipped with a XSelect CSH C18 reversed-phase HPLC column (Waters, 4.6 × 250 mm, 5.0-μm particle size). Isocratic elution was performed with acetonitrile/methanol/H_2_O (9:7:84, v/v) as mobile phase at a flow rate of 0.3 ml min^−1^ and fluorescence was monitored with a fluorescence detector (RF-10A XL, Shimadzu) at 372 nm for excitation and 456 nm for emission. For the assignment of individual peaks, the DMB-labelled Glyko sialic acid reference panel (Prozyme) was used.

### DMB-HPLC analysis of Golgi-confined Sia in HAP1 cells

For each cell type, HAP1 wt and HAP1^Δ*CASD1*^, 2 × 10^7^ cells were incubated in 100 μl PBS containing 15 mU sialidase isolated from *Arthrobacter ureafaciens* (EY Laboratories, Inc.) for 3 h at 37 °C to remove cell surface Sia. Cells were pelleted by centrifugation and lysed in 100 μl of PBS in a Precellys 24 homogenizer (Bertin Technologies) at 5,000 r.p.m. for 3 × 5 s. The microsomal fraction was obtained by centrifugation for 1 h at 100,000*g* and removal of the cytosolic fraction. The pellet was washed with PBS followed by centrifugation for 1 h at 100,000*g*, resuspended in 100 μl of 2 M propionic acid and incubated for 1 h at 80 °C to release bound Sia. After centrifugation for 1 h at 100,000*g*, the supernatant was lyophilized, derivatized with DMB and analysed by HPLC as described.

### LC-ESI-MS

MS analysis of peptides and DMB-labelled sugars was performed on a nanoACQUITY UPLC system (Waters) equipped with an analytical column (Waters, BEH130C18, 100 μm × 100 mm, 1.7-μm particle size) coupled online with an ESI Q-TOF (Q-TOF Ultima, Waters). DMB-labelled sugars dissolved in acetonitrile/methanol/H_2_O (9:7:84, v/v) and peptides dissolved in 2% acetonitrile and 0.1% formic acid were separated by reverse-phase chromatography using acetonitrile as eluent. MS spectra were recorded in positive reflection mode and analytes were automatically subjected to fragmentation (MS/MS). Spectra were analysed using MassLynx V4.1 software (Waters). MS/MS protein spectra were automatically analysed using the program ProteinLynx Global Server (Version 2.1, Waters).

### Ganglioside analysis by thin-layer chromatography

Gangliosides were extracted as described^19^. Briefly, 2 × 10^7^ cells were resuspended in chloroform/methanol (1:2, v/v), lysed for 15 min by sonication and centrifuged for 10 min at 3,000 r.p.m. Water was added to the supernatant to obtain a final ratio of chloroform/methanol/H_2_0 of 4:8:5 (v/v/v). After vortexing and centrifugation for 5 min at 4,800 r.p.m., the upper phase was recovered. The obtained ganglioside fraction was desalted on a Chromabond C18 cartridge (Macherey-Nagel) and evaporated to dryness under nitrogen gas. Gangliosides were resuspended in chloroform/methanol (1:2, v/v), spotted on high-performance TLC plates (Nano-DURASIL-20, 10 × 10 cm, Macherey-Nagel) and chromatographed for 30 min in chloroform/methanol/H_2_O (120:70:17, v/v/v) containing 0.02% CaCl_2_ (ref. 5). Plates were treated with 0.5% polyisobutyl methacrylate (Sigma Aldrich) in hexane and dried. After incubation in PBS followed by blocking with 1% BSA in PBS for 1 h at RT, immunostaining with enhanced chemiluminescence detection was performed using the following antibodies: Anti-CD60b mAb UM4D4 (5 μg ml^−1^; Santa Cruz cat. Sc-32269); anti-CD60c mAb U5 (40 μg ml^−1^; kindly provided by Reinhard Schwartz-Albiez); anti-CD60a mAb R24 (10 μg ml^−1^; purified from cell culture supernatant of hybridoma cells by Protein A affinity chromatography); goat anti-mouse IgG HRP conjugate (1:5,000; Southern Biotech cat. 1010-05); or goat anti-mouse IgM HRP conjugate (1:5,000; Southern Biotech cat. 1021-05). To induce migration of *O*-acetyl groups from the C7 to the C9 position of Sia, TLC plates were treated with 0.1 M glycine/NaOH pH 9.7 for 30 min at 37 °C (ref. 5) before blocking with 1% BSA in PBS. 7-*O*- and 9-*O*-acetylated GD3 used as standards were purified from bovine buttermilk by Bernhard Kniep^25^ and have been generously provided by Reinhard Schwartz-Albiez.

## Additional information

**How to cite this article:** Baumann, A-M.T. *et al.* 9-*O*-Acetylation of sialic acids is catalysed by CASD1 *via* a covalent acetyl-enzyme intermediate. *Nat. Commun.* 6:7673 doi: 10.1038/ncomms8673 (2015).

## Supplementary information

Supplementary Information Supplementary Figures 1-10 and Supplementary References (PDF 5068 kb)

## References

[1] Varki, A. & Schauer, R. in *Essentials of Glycobiology* 2nd edn eds Varki A.et al. 199–217Cold Spring Harbor Laboratory Press (2009).

[2] Kelm S, Schauer R, Manuguerra JC, Gross HJ, Crocker PR (1994). Modifications of cell surface sialic acids modulate cell adhesion mediated by sialoadhesin and CD22. Glycoconj. J..

[3] Padler-Karavani V (2014). Rapid evolution of binding specificities and expression patterns of inhibitory CD33-related Siglecs in primates. FASEB J..

[4] Shi WX, Chammas R, Varki NM, Powell L, Varki A (1996). Sialic acid 9-*O*-acetylation on murine erythroleukemia cells affects complement activation, binding to I-type lectins, and tissue homing. J. Biol. Chem..

[5] Schauer R, Srinivasan GV, Wipfler D, Kniep B, Schwartz-Albiez R (2011). *O*-Acetylated sialic acids and their role in immune defense. Adv. Exp. Med. Biol..

[6] Mandal, C., Schwartz-Albiez, R. & Vlasak, R. Functions and biosynthesis of *O*-acetylated sialic acids. *Top. Curr. Chem* doi: 10.1007/128_2011_310 (2012). 10.1007/128_2011_310 PMC7120186 22371169

[7] Rogers GN, Herrler G, Paulson JC, Klenk H-D (1986). Influenza C virus uses 9-*O-*acetyl-*N*-acetylneuraminic acid as a high affinity receptor determinant for attachment to cells. J. Biol. Chem..

[8] De Groot RJ (2006). Structure, function and evolution of the hemagglutinin-esterase proteins of corona- and toroviruses. Glycoconj. J..

[9] Vlasak R, Luytjes W, Leider J, Spaan W, Palese P (1988). The E3 protein of bovine coronavirus is a receptor-destroying enzyme with acetylesterase activity. J. Virol..

[10] Müller J, Nitschke L (2014). The role of CD22 and Siglec-G in B-cell tolerance and autoimmune disease. Nat. Rev. Rheumatol..

[11] Sjoberg ER, Powell LD, Klein A, Varki A (1994). Natural ligands of the B cell adhesion molecule CD22beta can be masked by 9-*O*-acetylation of sialic acids. J. Cell Biol..

[12] Cariappa A (2009). B cell antigen receptor signal strength and peripheral B cell development are regulated by a 9-*O*-acetyl sialic acid esterase. J. Exp. Med..

[13] Surolia I (2010). Functionally defective germline variants of sialic acid acetylesterase in autoimmunity. Nature.

[14] Chellappa V (2013). M89V sialic acid acetyl esterase (SIAE) and all other non-synonymous common variants of this gene are catalytically normal. PLoS ONE.

[15] Santiago MF, Costa MR, Mendez-Otero R (2004). Immunoblockage of 9-*O*-acetyl GD3 ganglioside arrests the *in vivo* migration of cerebellar granule neurons. J. Neurosci..

[16] Ribeiro-Resende VT (2007). Ganglioside 9-*O*-acetyl GD3 expression is upregulated in the regenerating peripheral nerve. Neuroscience.

[17] Ribeiro-Resende VT (2010). Involvement of 9-*O*-Acetyl GD3 ganglioside in *Mycobacterium leprae* infection of Schwann cells. J. Biol. Chem..

[18] De Maria R (1997). Requirement for GD3 ganglioside in CD95- and ceramide-induced apoptosis. Science.

[19] Malisan F (2002). Acetylation suppresses the proapoptotic activity of GD3 ganglioside. J. Exp. Med..

[20] Birks SM (2011). Targeting the GD3 acetylation pathway selectively induces apoptosis in glioblastoma. Neuro Oncol..

[21] Kniep B (2006). 9-*O*-acetyl GD3 protects tumor cells from apoptosis. Int. J. Cancer.

[22] Mukherjee K (2008). *O*-acetylation of GD3 prevents its apoptotic effect and promotes survival of lymphoblasts in childhood acute lymphoblastic leukaemia. J. Cell. Biochem..

[23] Parameswaran R (2013). *O*-acetylated *N*-acetylneuraminic acid as a novel target for therapy in human pre-B acute lymphoblastic leukemia. J. Exp. Med..

[24] Erdmann M (2006). Differential surface expression and possible function of 9-*O*- and 7-*O-*acetylated GD3 (CD60 b and c) during activation and apoptosis of human tonsillar B and T lymphocytes. Glycoconj. J..

[25] Kniep B (1995). 7-*O*-acetyl-GD3 in human T-lymphocytes is detected by a specific T-cell-activating monoclonal antibody. J. Biol. Chem..

[26] Varki A, Diaz S (1985). The transport and utilization of acetyl coenzyme A by rat liver golgi vesicles. *O*-acetylated sialic acids are a major product. J. Biol. Chem..

[27] Higa HH, Butor C, Diaz S, Varki A (1989). *O*-Acetylation and De-*O*-acetylation of sialic acids. *O*-acetylation of sialic acids in the rat liver Golgi apparatus involves an acetyl intermediate and essential histidine and lysine residues—a transmembrane reaction?. J. Biol. Chem..

[28] Ogura K (1996). Cloning and expression of cDNA for *O*-acetylation of GD3 Ganglioside. Biochem. Biophys. Res. Commun..

[29] Kanamori A (1997). Expression cloning and characterization of a cDNA encoding a novel membrane protein required for the formation of *O*-acetylated ganglioside: a putative acetyl-CoA transporter. Proc. Natl Acad. Sci. USA.

[30] Shi W-X, Chammas R, Varki A (1998). Induction of sialic acid 9-*O*-acetylation by diverse gene products: implications for the expression cloning of sialic acid *O*-acetyltransferases. Glycobiology.

[31] Diaz S, Higa HH, Hayes BK, Varki A (1989). *O*-Acetylation and de-*O*-acetylation of sialic acids. 7- and 9-*O*-acetylation of α-2,6-linked sialic acids on endogenous N-linked glycans in rat liver Golgi vesicles. J. Biol. Chem..

[32] Lrhorfi LA, Srinivasan GV, Schauer R (2007). Properties and partial purification of sialate-*O*-acetyltransferase from bovine submandibular glands. Biol. Chem..

[33] Shen Y (2002). Characterization of the Sialate-7(9)-*O*-acetyltransferase from the microsomes of human colonic mucosa. Biol. Chem..

[34] Butor C, Diaz S, Varki A (1993). High level *O*-acetylation of sialic acids on N-linked oligosaccharides of rat liver membranes. Differential subcellular distribution of 7- and 9-*O*-acetyl groups and of enzymes involved in their regulation. J. Biol. Chem..

[35] Arming S (2011). The human Cas1 protein: a sialic acid-specific *O*-acetyltransferase?. Glycobiology.

[36] Janbon G, Himmelreich U, Moyrand F, Improvisi L, Dromer F (2001). Cas1p is a membrane protein necessary for the *O*-acetylation of the *Cryptococcus neoformans* capsular polysaccharide. Mol. Microbiol..

[37] Dumermuth E, Beuret N, Spiess M, Crottet P (2002). Ubiquitous 9-*O*-acetylation of sialoglycoproteins restricted to the Golgi complex. J. Biol. Chem..

[38] Akoh CC, Lee G-C, Liaw Y-C, Huang T-H, Shaw J-F (2004). GDSL family of serine esterases/lipases. Prog. Lipid Res..

[39] Anantharaman V, Aravind L (2010). Novel eukaryotic enzymes modifying cell-surface biopolymers. Biol. Direct.

[40] Velasco A (1993). Cell type-dependent variations in the subcellular distribution of α-mannosidase I and II. J. Cell Biol..

[41] Rosenthal PB (1998). Structure of the haemagglutinin-esterase-fusion glycoprotein of influenza C virus. Nature.

[42] Zeng Q, Langereis MA, van Vliet AL, Huizinga EG, de Groot RJ (2008). Structure of coronavirus hemagglutinin-esterase offers insight into corona and influenza virus evolution. Proc. Natl Acad. Sci. USA.

[43] Rangarajan ES (2011). Structural and enzymatic characterization of NanS (YjhS), a 9-*O*-Acetyl *N*-acetylneuraminic acid esterase from *Escherichia coli* O157:H7. Protein Sci..

[44] Jarvis DL, Finn EE (1996). Modifying the insect cell N-glycosylation pathway with immediate early baculovirus expression vectors. Nat. Biotechnol..

[45] Klein A (1997). New sialic acids from biological sources identified by a comprehensive and sensitive approach: liquid chromatography-electrospray ionization-mass spectrometry (LC-ESI-MS) of SIA quinoxalinones. Glycobiology.

[46] Vlasak R, Luytjes W, Spaan W, Palese P (1988). Human and bovine coronaviruses recognize sialic acid-containing receptors similar to those of influenza C viruses. Proc. Natl Acad. Sci USA.

[47] Padler-Karavani V (2012). Cross-comparison of protein recognition of sialic acid diversity on two novel sialoglycan microarrays. J. Biol. Chem..

[48] Hsu PD, Lander ES, Zhang F (2014). Development and applications of CRISPR-Cas9 for genome engineering. Cell.

[49] Haraguchi M (1994). Isolation of GD3 synthase gene by expression cloning of GM3 α-2,8-sialyltransferase cDNA using anti-GD2 monoclonal antibody. Proc. Natl Acad. Sci USA.

[50] Kniep B, Flegel WA, Northoff H, Rieber EP (1993). CDw60 glycolipid antigens of human leukocytes: structural characterization and cellular distribution. Blood.

[51] Kniep B, Peter-Katalinić J, Flegel W, Northoff H, Rieber EP (1992). CDw 60 antibodies bind to acetylated forms of ganglioside GD3. Biochem. Biophys. Res. Commun..

[52] Kuhn B (2013). The structure of human α-2,6-sialyltransferase reveals the binding mode of complex glycans. Acta Crystallogr. D. Biol. Crystallogr..

[53] Shi W-X, Chammas R, Varki A (1996). Linkage-specific action of endogenous sialic acid *O*-acetyltransferase in Chinese hamster ovary cells. J. Biol. Chem..

[54] Corfield AP, Ferreira do Amaral C, Wember M, Schauer R (1976). The metabolism of *O*-acyl-*N*-acylneuraminic acids. Biosynthesis of *O*-acylated sialic acids in bovine and equine submandibular glands. Eur. J. Biochem..

[55] Shen Y, Tiralongo J, Kohla G, Schauer R (2004). Regulation of sialic acid *O*-acetylation in human colon mucosa. Biol. Chem..

[56] Rauvolfova J, Venot A, Boons G-J (2008). Chemo-enzymatic synthesis of C-9 acetylated sialosides. Carbohydr. Res..

[57] Varki A, Diaz S (1984). The release and purification of sialic acids from glycoconjugates: Methods to minimize the loss and migration of *O*-acetyl groups. Anal. Biochem..

[58] Kamerling JP (1987). Migration of *O*-acetyl groups in *N,O*-acetylneuraminic acids. Eur. J. Biochem..

[59] Vandamme-Feldhaus V, Schauer R (1998). Characterization of the enzymatic 7-*O*-acetylation of sialic acids and evidence for enzymatic *O*-acetyl migration from C-7 to C-9 in bovine submandibular gland. J. Biochem..

[60] Wipfler D (2011). Differentially regulated expression of 9-*O*-acetyl GD3 (CD60b) and 7-*O*-acetyl-GD3 (CD60c) during differentiation and maturation of human T and B lymphocytes. Glycobiology.

[61] Bergfeld AK (2009). The polysialic acid-specific *O*-acetyltransferase OatC from *Neisseria meningitidis* serogroup C evolved apart from other bacterial sialate *O*-acetyltransferases. J. Biol. Chem..

[62] Moynihan PJ, Clarke AJ (2014). Mechanism of action of peptidoglycan *O*-acetyltransferase B involves a Ser-His-Asp catalytic triad. Biochemistry.

[63] Williams AH (2014). Visualization of a substrate-induced productive conformation of the catalytic triad of the *Neisseria meningitidis* peptidoglycan *O*-acetylesterase reveals mechanistic conservation in SGNH esterase family members. Acta Crystallogr. D. Biol. Crystallogr..

[64] Bernard E (2011). Characterization of *O*-acetylation of *N*-acetylglucosamine: a novel structural variation of bacterial peptidoglycan. J. Biol. Chem..

[65] Riley LM (2013). Structural and functional characterization of *Pseudomonas aeruginosa* AlgX: role of AlgX in alginate acetylation. J. Biol. Chem..

[66] Kelley LA, Mezulis S, Yates CM, Wass MN, Sternberg MJE (2015). The Phyre2 web portal for protein modeling, prediction and analysis. Nat. Protoc..

[67] Zheng Q (2014). Precise gene deletion and replacement using the CRISPR/Cas9 system in human cells. Biotechniques.

[68] Eckhardt M, Gotza B, Gerardy-Schahn R (1999). Membrane topology of the mammalian CMP-sialic acid transporter. J. Biol. Chem..

[69] Mawhinney TP, Chance DL (1994). Hydrolysis of sialic acids and *O*-acetylated sialic acids with propionic acid. Anal. Biochem..

[70] Hara S (1989). Determination of mono-*O*-acetylated *N*-acetylneuraminic acids in human and rat sera by fluorometric high-performance liquid chromatography. Anal. Biochem..

