# Supplementary material for: 9-O-Acetylation of sialic acids is catalysed by CASD1 via a covalent acetyl-enzyme intermediate
Source: Nat Commun. 2015 Jul 14;6:7673. doi: 10.1038/ncomms8673 (PMC4510713; doi:10.1038/ncomms8673)
Supplement: Supplementary Information — Supplementary Figures 1-10 and Supplementary References (PDF 5068 kb) [file 41467_2015_BFncomms8673_MOESM248_ESM.pdf]

# Supplementary Figures

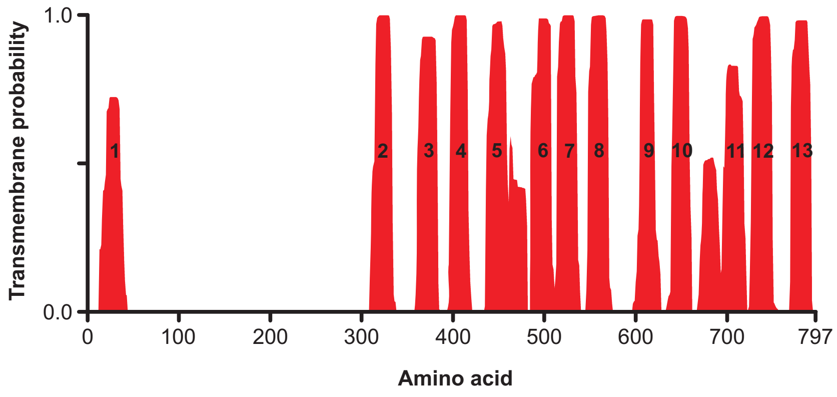


**Supplementary Figure 1. Prediction of transmembrane helices of human CASD1.** Transmembrane helices were predicted using the program TMHMM 2.0[1].

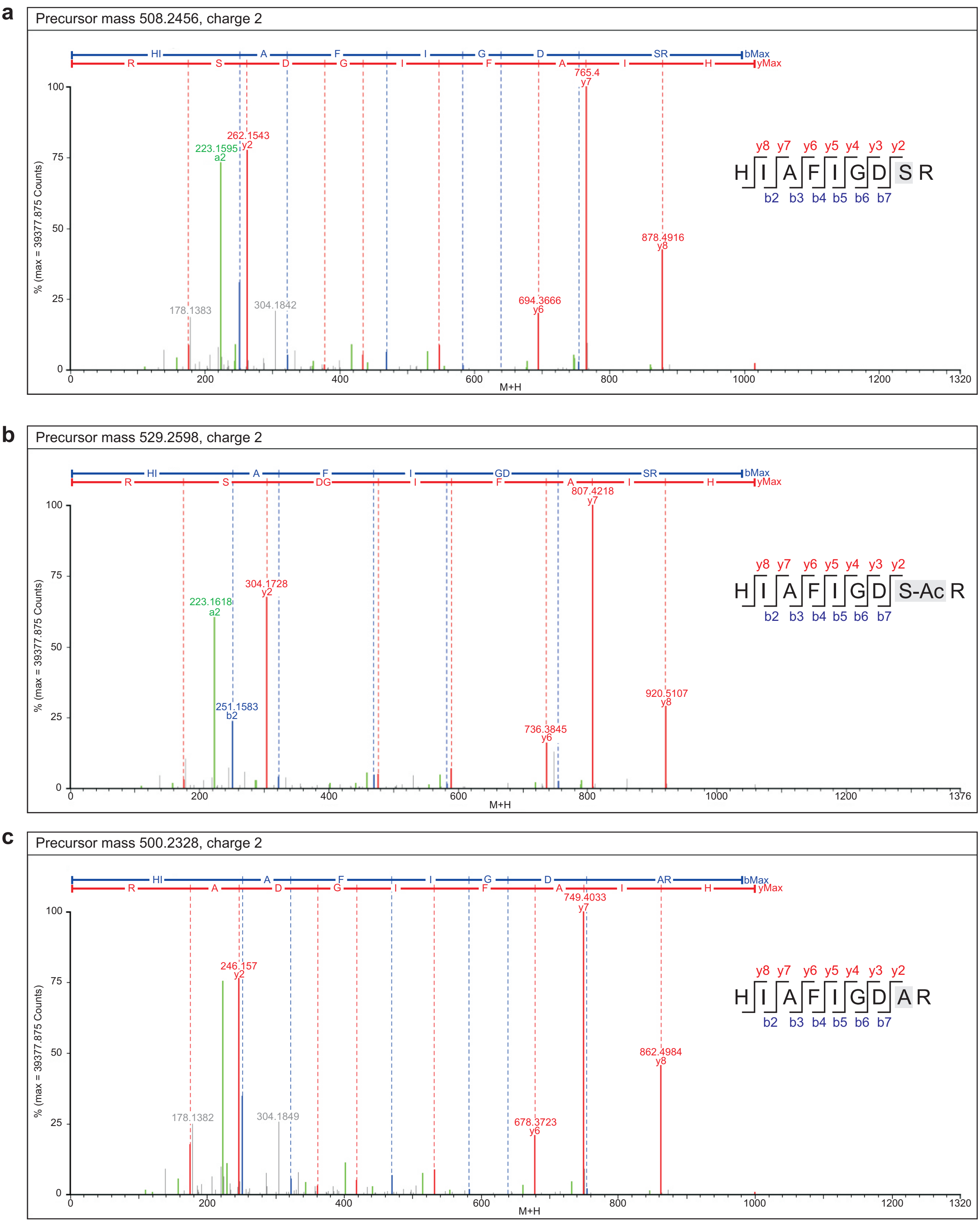


**Supplementary Figure 2. Identification of an acetyl-enzyme intermediate involving S94 of CASD1.** The CASD1 peptide for which an acetyl adduct had been observed (Fig. 2f) was analysed by peptide sequencing using LC-ESI-MS/MS. (**a**,**b**) Tandem MS spectra of the doubly charged peptide in non-acetylated (*m/z* = 508.25) and acetylated (*m/z* = 529.26) form obtained from sCASD1-wt incubated with acetyl-CoA. (**c**) Tandem MS spectra of the doubly charged peptide (*m/z* = 500.23) obtained from sCASD1-S94A incubated with acetyl-CoA. MS/MS spectra were analysed using the program ProteinLynx Global Server (Version 2.1, Waters). The fragmentation patterns of the respective peptides are given on the right and detected C-terminal (y-series) and N-terminal (b-series) ions are indicated on the sequence.

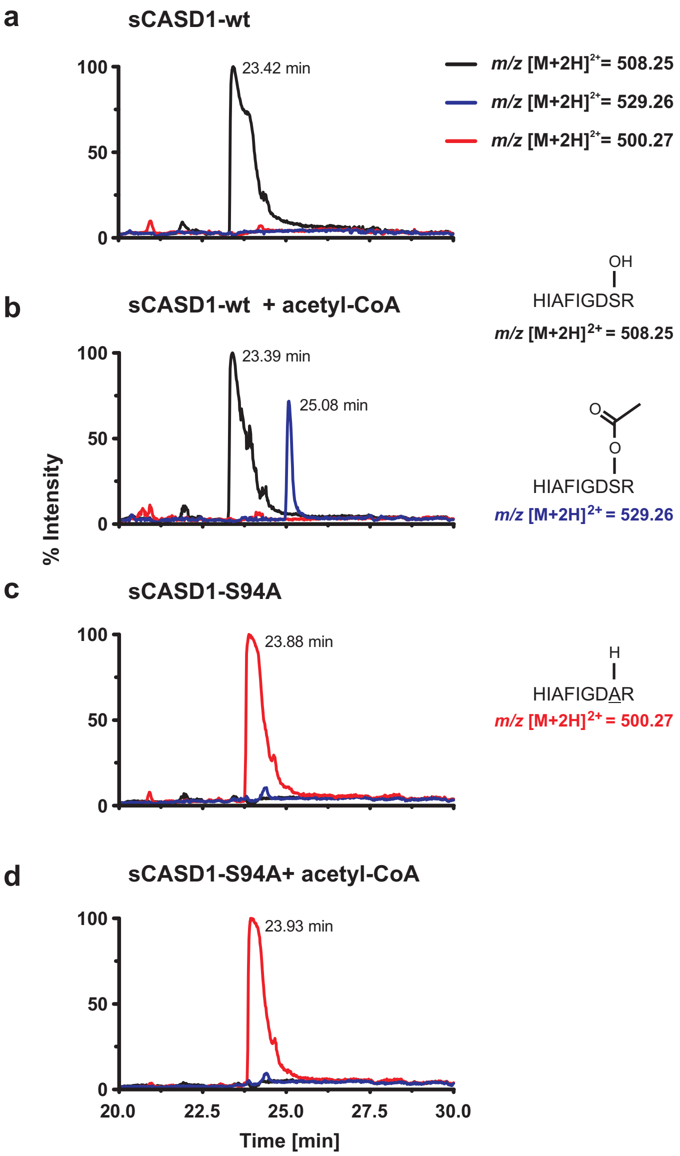


**Supplementary Figure 3. LC-ESI-MS analysis of the acetyl-enzyme intermediate of CASD1.** Purified sCASD1-wt and sCASD1-S94A were incubated with or without acetyl-CoA. After separation by SDS-PAGE and tryptic digest of the excised protein bands, each sample was analysed by LC-ESI-MS. Theoretical *m/z* values are given for the doubly charged tryptic peptides containing the active site serine S94 or the corresponding S94A mutation. Extracted ion chromatograms (EICs) were generated by searching the chromatograms for the respective ions with the theoretical *m/z* values for the 2-fold charged tryptic peptide of sCASD1-wt (87-HIAFIGDSR-95, *m/z* = 508.25), the serine *O*-acetylated peptide (87-HIAFIGDS(ac)R-95, *m/z* = 529.26) and the peptide of sCASD1-S94A (87-HIAFIGDAR-95, *m/z* = 500.27). (**a**) In the absence of acetyl-CoA, only one peak was observed for sCASD1-wt in EICs corresponding to the unmodified peptide (*m/z* = 508.25, black line). (**b**) Addition of acetyl-CoA to the reaction mixture led to the identification of a further peak in the EIC for *m/z* = 529.26 (blue line), corresponding to the mass of the serine *O*-acetylated peptide. (**c,d**) Analysis of sCASD1-S94A revealed a peak in the EIC corresponding to the unmodified peptide with the *m/z* value of 500.27 (red line). In the reaction mixtures containing sCASD1-S94A, no masses for peptides corresponding to sCASD1-wt were observed. Spectra shown for the respective samples (Fig. 2f) were generated from EICs by accumulation of scans ± 0.1 min around peak maxima. The identity of the peptides was confirmed by LC-ESI-MS/MS (Supplementary Fig. 2).

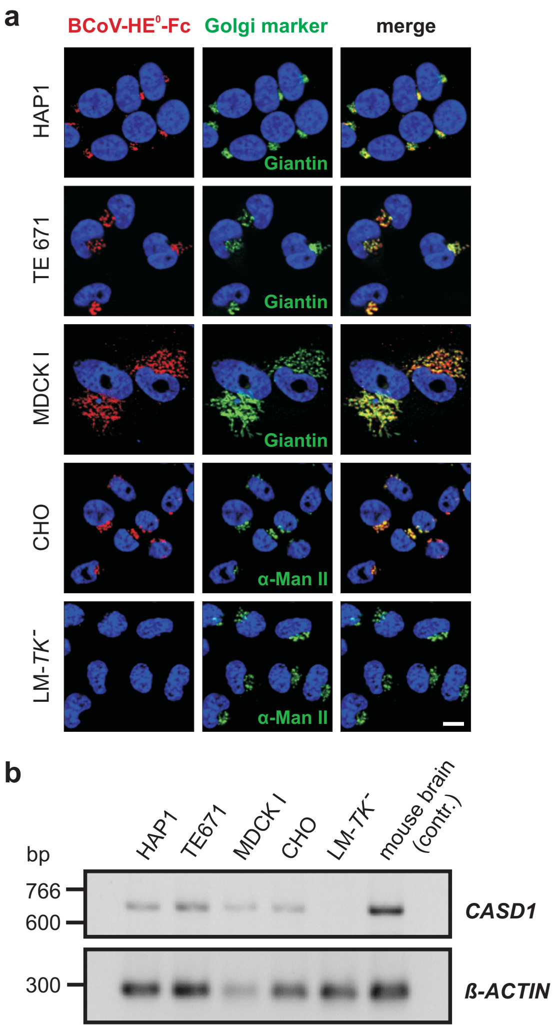


**Supplementary Figure 4. Golgi-localized 9-*O*-acetylated sialoglycoconjugates in mammalian cell lines.** (**a**) Triton-permeabilized HAP1[2] (human), TE671[3,4] (human), MDCK I[5] (canine), CHO[6] (hamster) and LM-*TK*[-7] (murine) cells were co-stained with BCoV-HE$^0$-Fc to detect 9-*O*-acetylated Sia and an antibody recognizing either Giantin (human and canine cell lines) or α-mannosidase II (α-Man II, murine and hamster cell lines). Nuclei were counterstained with DAPI (blue). Scale bar: 10 µm. (**b**) Analysis of *CASD1* expression by RT-PCR. *CASD1* transcripts were amplified using exon 2 and exon 8 specific forward and reverse primers, respectively. RNA isolated from perinatal mouse brain was used as positive control (contr.). *β-ACTIN* transcripts were amplified as internal control. PCR products were separated on a 2.5% agarose gel and stained with ethidium bromide. TE671 and MDCK I cells were kindly provided by Thorsten Pietsch[3] and Georg Herrler[5], respectively. Please note that TE671 was originally described as human medulloblastoma line, but was later demonstrated to be a sub-line of the human rhabdomyosarcoma cell line RD[4]. TE671 were part of our screen for SOAT-negative cells and are shown here as one example of a cell line with Golgi-confined 9-*O*-acetylated sialoglycans.

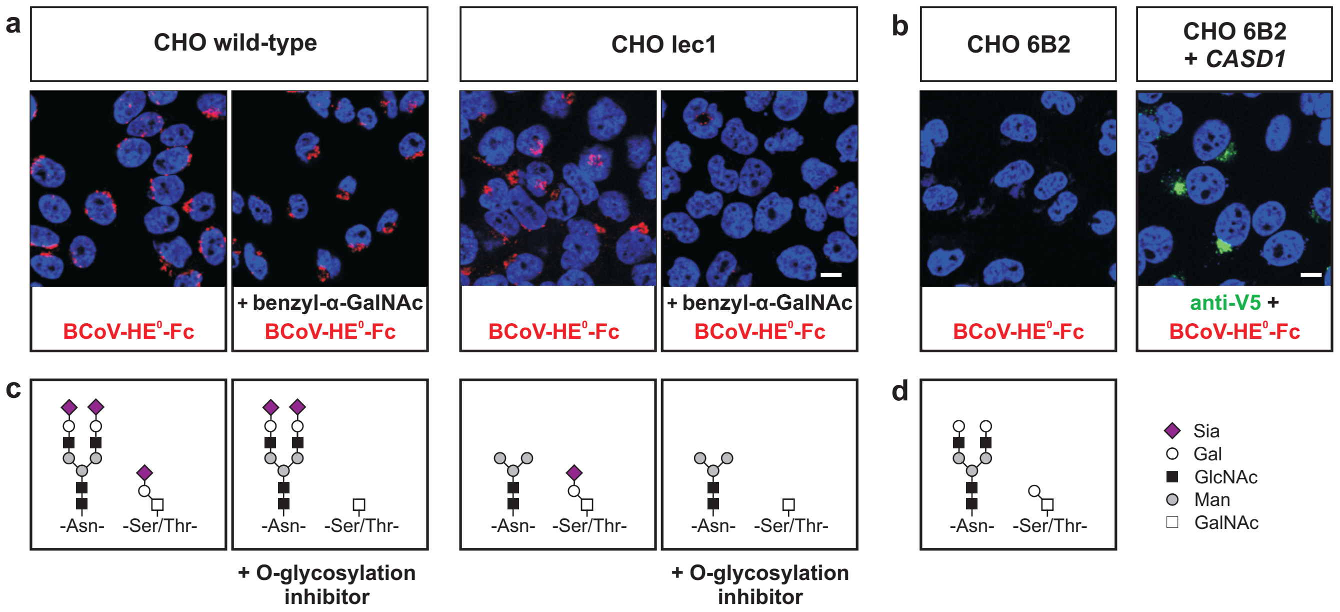


**Supplementary Figure 5. Golgi-confined 9-*O*-acetylated Sia is attached to N- and O-glycans.** (**a**) Triton-permeabilized CHO wild-type and CHO lec1 cells[8] were stained with BCoV-HE$^{0}$-Fc to detect 9-*O*-acetylated sialoglycans. Parallel stainings were performed with cells in which the synthesis of mucin-type O-glycans was blocked by treatment with benzyl-α-GalNAc. This structural analogue of GalNAc-Ser/Thr acts as a competitive inhibitor of O-glycan extension by serving as a decoy acceptor for enzymes involved in these extensions[9,10]. CHO lec1 cells lack complex and hybrid-type N-glycans due to a defect in the *N*-acetylglucosaminyltransferase I gene[8]. Nuclei were counterstained with DAPI (blue). (**b**) As a control for BCoV-HE$^{0}$-Fc staining, Triton-permeabilized CHO 6B2 cells that produce asialo-glycoconjugates due to a defect in the Golgi CMP-Sia-transporter[11] were stained with BCoV-HE$^{0}$-Fc. Moreover, CHO 6B2 cells transfected with a plasmid encoding CASD1 with an N-terminal V5-tag (+ *CASD1*) were co-stained with anti-V5 (green) and BCoV-HE$^{0}$-Fc (red). Nuclei were counterstained with DAPI (blue). Scale bar: 10 µm. (**c,d**) Scheme depicting variations in the glycosylation pattern. Differences between wild-type and mutant cells as well as before and after treatment with the O-glycosylation inhibitor benzyl-α-GalNAc are illustrated for a sialylated biantennary N-glycan and a sialylated core 1 O-glycan. CHO lec1 cells were kindly provided by Pamela Stanley.

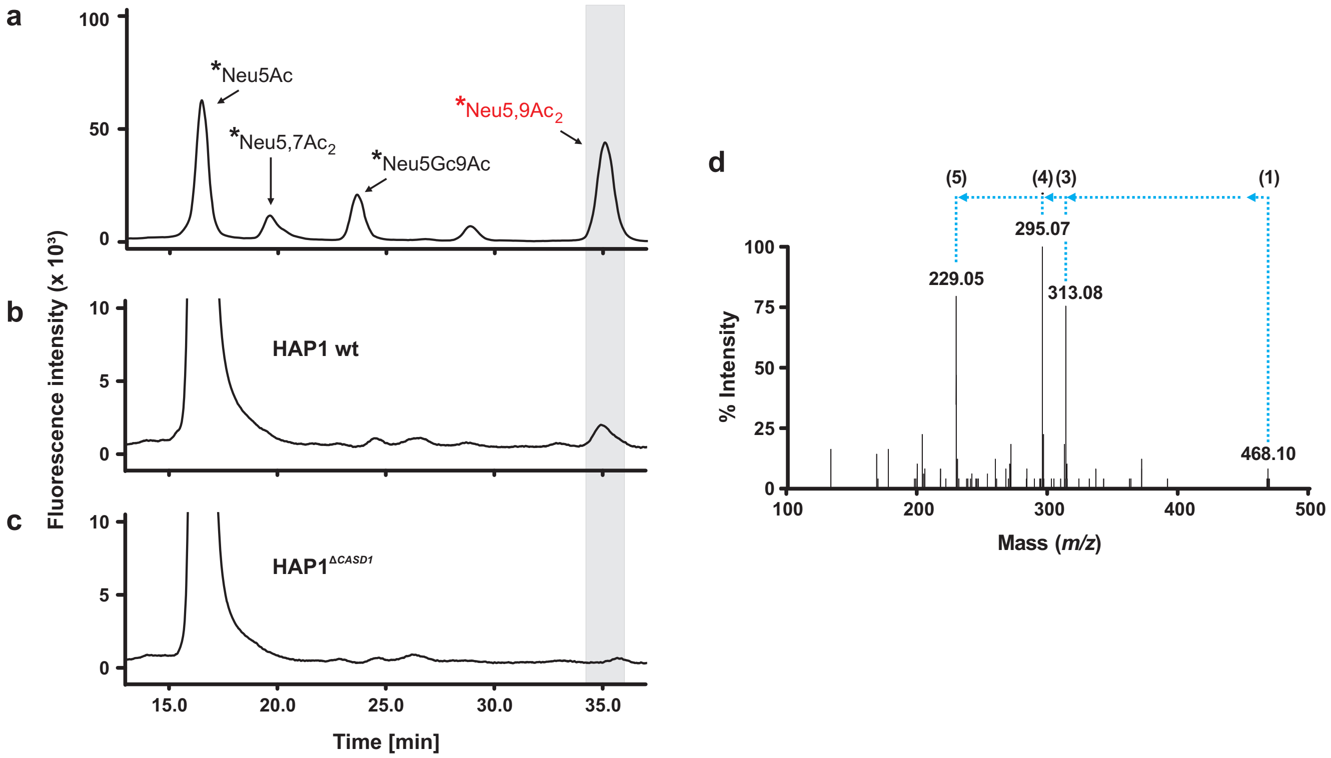


**Supplementary Figure 6. Analysis of Golgi-confined Sia in HAP1 cells by DMB-HPLC and LC-ESI-MS/MS.** (**a**) HPLC elution profile of the DMB-derivatized Sia reference panel. Asterisks denote that the indicated Sia species are labelled with DMB. (**b**,**c**) HPLC elution profiles of DMB-derivatized Sia obtained from microsomal fractions of 2 x $10^7$ HAP1 wild-type (wt) and HAP1$^{\Delta CASD1}$ cells (see Methods Section for details). The retention time corresponding to DMB-Neu5,9Ac$_2$ of the reference panel is highlighted by a grey box. (**d**) LC-ESI-MS/MS analysis. The peak material from HAP1 wt and HAP1$^{\Delta CASD1}$ cells eluting at the retention time of DMB-Neu5,9Ac$_2$ was collected and subjected to LC-ESI-MS/MS. Ions at *m/z* 468.16 ($[M+H]^+$), corresponding to DMB-Neu5,9Ac$_2$, were only obtained for the material collected from (b) and subsequent fragmentation revealed the three ions characteristic for DMB-Neu5,9Ac$_2$ (*m/z* $[M+H]^+$: 313, 295, 229)[12]. Numbers in brackets above the spectrum refer to the structures depicted in Figure 3f.

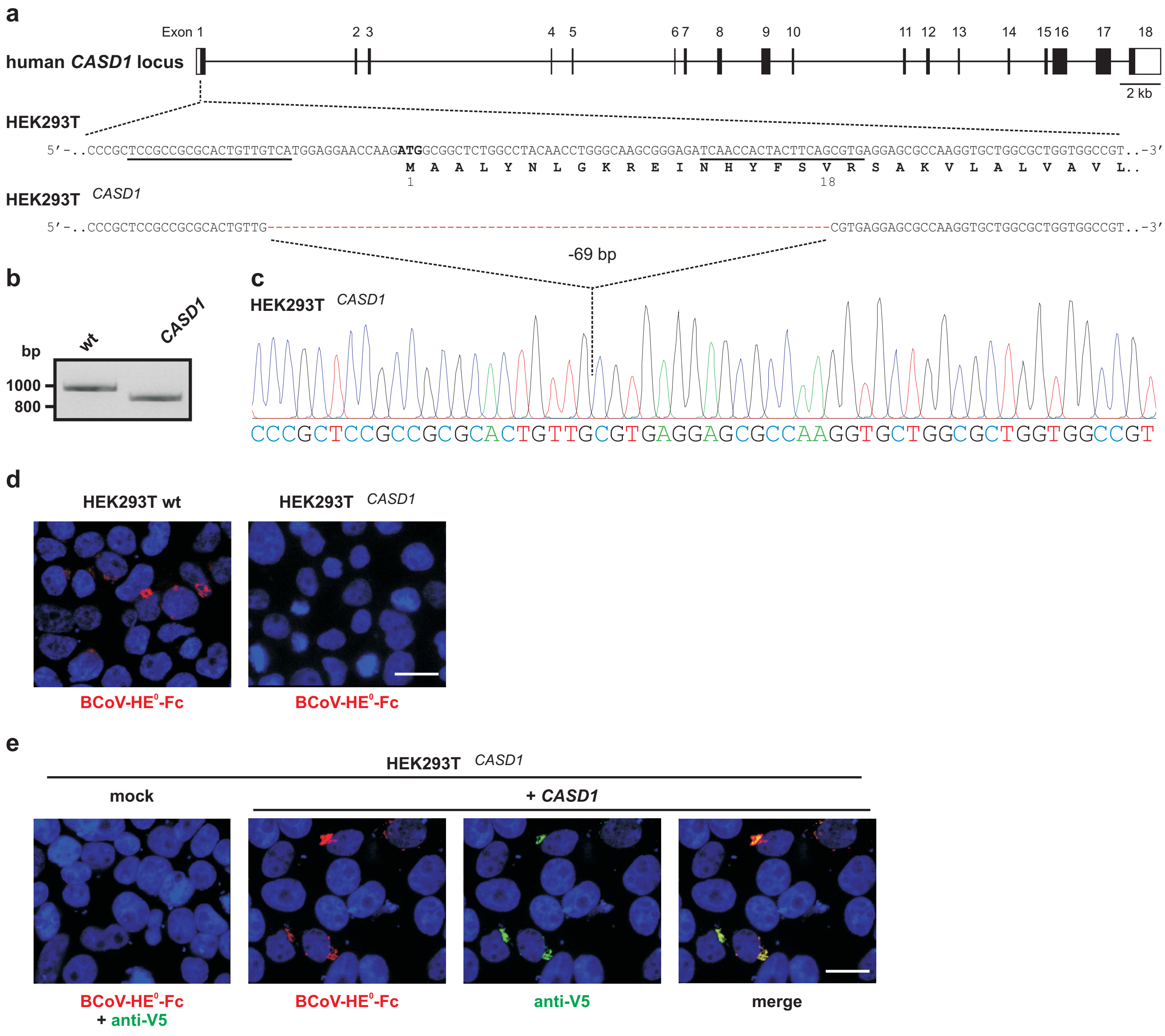


**Supplementary Figure 7. *CASD1* knock-out results in loss of Sia 9-*O*-acetylation in HEK293T cells.** (**a**) Schematic representation of the human *CASD1* locus, showing the target sites used for CRISPR/Cas-mediated genome editing in HEK293T cells. A two-target editing approach was used to make precise 69-bp deletions at the 5' end of exon 1 in both alleles[13], resulting in deletion of the Kozak sequence and the sequence encoding amino acid residues 1 through 18. (**b**) PCR analysis of genomic DNA from parental HEK293T cells (wt) and HEK293T$^{\Delta CASD1}$ cells (Δ*CASD1*), showing a deletion in *CASD1* exon 1 in the latter cells. (**c**) Sequence analysis of the PCR amplicon from HEK293T$^{\Delta CASD1}$ cells, showing the deletion of nucleotide residues -16 through 53 of *CASD1* exon 1. (**d**) Immunofluorescence analysis of Triton-X-100-permeabilized HEK293T wt and HEK293T$^{\Delta CASD1}$ cells stained with BCoV-HE$^0$-Fc. (**e**) Complementation of HEK293T$^{\Delta CASD1}$ cells with *CASD1* cDNA restores sialate 9-*O*-acetylation. HEK293T$^{\Delta CASD1}$ cells were transiently transfected with empty vector (mock) or with a plasmid encoding V5-tagged CASD1 (+ *CASD1*). Permeabilized cells were co-stained with BCoV-HE$^0$-Fc (red) and anti-V5 mAb (green). Nuclei were counterstained with DAPI (blue). Scale bar: 10 µm.

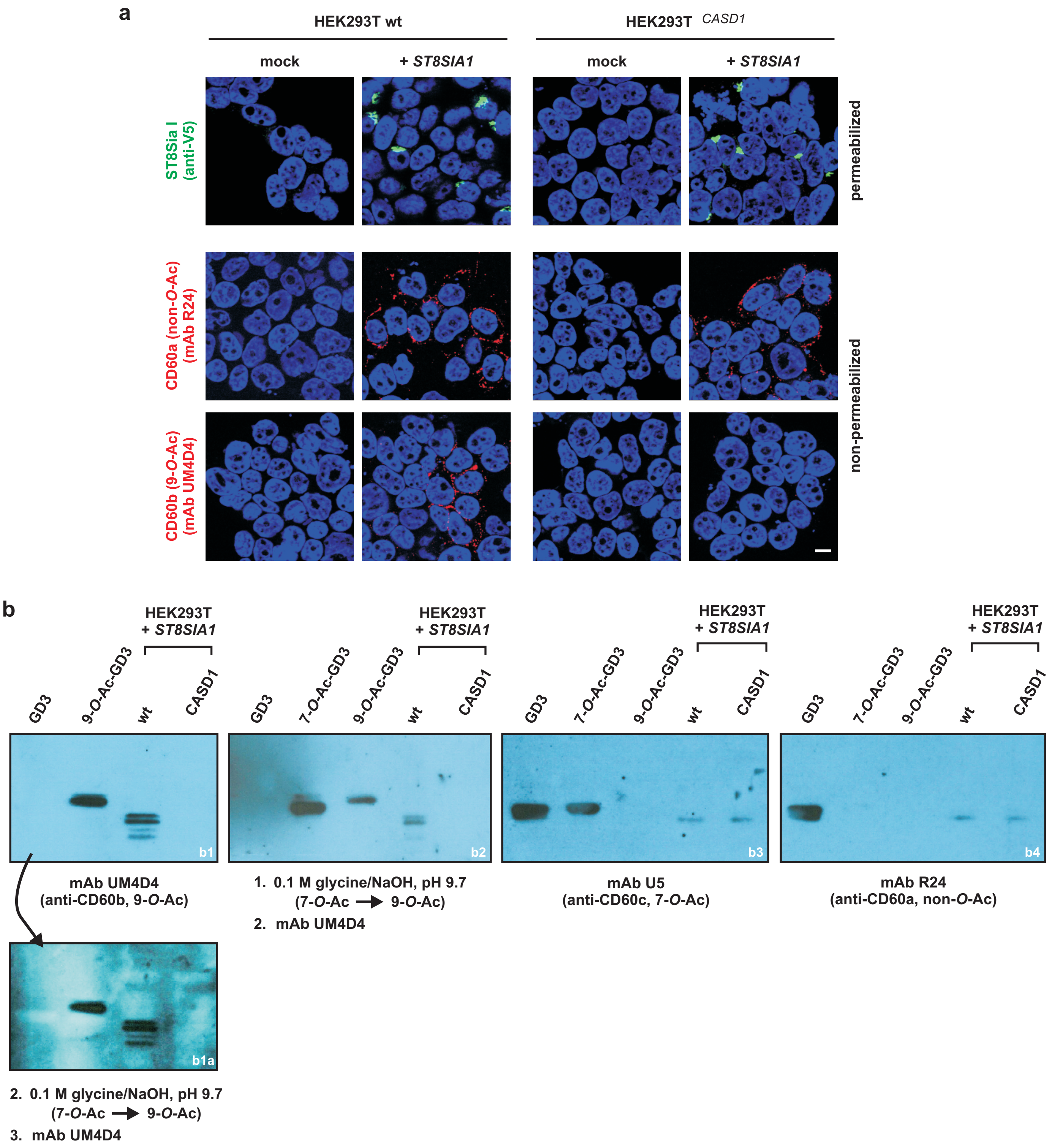


**c**

| | | |
|---|---|---|
| 9-*O*-Ac-GD3 | III$^{8}$- -Neu5,9Ac$_{2}$,II$^{3}$- -Neu5Ac-LacCer | **9-*O*-Ac**-Sia 2-8-Sia 2-3-Gal 1-4Glc 1-1'-Cer |
| 9-*O*-Ac-DSPG | V$^{8}$- -Neu5,9Ac$_{2}$,IV$^{3}$- -Neu5Ac-nLc$_{4}$Cer | **9-*O*-Ac**-Sia 2-8-Sia 2-3-Gal 1-4GlcNAc 1-3Gal 1-4Glc 1-1'-Cer |
| 9-*O*-Ac-DSHC | VII$^{8}$- -Neu5,9Ac$_{2}$,VI$^{3}$- -Neu5Ac-nLc$_{6}$Cer | **9-*O*-Ac**-Sia 2-8-Sia 2-3-Gal 1-4GlcNAc 1-3Gal 1-4GlcNAc 1-3Gal 1-4Glc 1-1'-Cer |

**Supplementary Figure 8. CASD1 mediates 9-*O*-acetylation of disialo gangliosides in HEK293T cells.** (**a**) HEK293T wt and HEK293T$^{\Delta CASD1}$ cells were transiently transfected with either empty vector (mock) or *ST8SIA1* cDNA (+ *ST8SIA1*). Triton-permeabilized cells (upper panel) were stained with anti-V5 mAb for the detection of V5-tagged ST8Sia I. Non-permeabilized cells were stained with anti-CD60a mAb R24 (middle panel) or anti-CD60b mAb UM4D4 (lower panel). Nuclei were counterstained with DAPI (blue). Scale bar: 10 µm.

[continued on next page]

**Supplementary Figure 8.** [continued]

(**b**) The higher transfection efficiency of HEK293T compared to HAP1 cells allowed an additional characterization of the generated *O*-acetylated gangliosides by thin-layer chromatography (TLC). Gangliosides extracted from 2 x $10^7$ HEK293T wt and HEK293T$^{\Delta CASD1}$ cells were separated by TLC and either stained with anti-CD60b mAb UM4D4 (b1, b2), anti-CD60c mAb U5 (b3) or anti-CD60a mAb R24 (b4). Purified GD3, 7-*O*-Ac-GD3 and 9-*O*-Ac-GD3 were used as standards. Upon ST8Sia I expression, mAb UM4D4-positive bands were observed in ganglioside extracts of HEK293T wt but not of HEK293T$^{\Delta CASD1}$ cells (b1). The mobility of these bands on the TLC plate indicated the formation of 9-*O*-acetylated gangliosides of the neolacto series, with the upper band co-migrating with non-*O*-acetylated GD3 as described for 9-*O*-acetylated disialo neolactotetraosylceramide[14]. Pre-treatment of TLC plates with mild alkali to induce a shift of *O*-acetyl groups from C7 to C9[14,15] did not result in the appearance of additional UM4D4-positive bands in the HEK293T samples (b2). This was also true for the plate shown in (b1) after additional mild alkali treatment and a second round of mAb UM4D4 staining (b1a), although this procedure was accompanied by high background staining. Immunostaining with anti-CD60c mAb U5 (b3) revealed binding of this mAb to the 7-*O*-acetylated GD3 standard, but also demonstrated cross-reactivity with non-modified GD3 as described previously[16]. Similarly, a slight cross-reactivity of anti-GD3 mAb R24 with 7-*O*-acetylated GD3 was observed (b4) as noted before[16]. However, based on their migration compared to the standards, the single bands detected with mAb U5 (b3) and mAb R24 (b4) in ganglioside extracts from ST8Sia I expressing HEK293T wt and HEK293T$^{\Delta CASD1}$ cells can be assigned to non-*O*-acetylated GD3. **(c)** Structural composition of 9-*O*-acetylated GD3 and similar gangliosides of the neolacto series that carry a terminal 9-*O*-acetylated disialo epitope recognized by anti-CD60b mAb UM4D4[14]. Commonly used abbreviations and ganglioside names according to IUPAC nomenclature are given in the first and second column, respectively. 9-*O*-Ac, 9-*O*-acetylated; DSPG, disialo paragloboside; DSHC, disialo neolactohexaosylceramide.

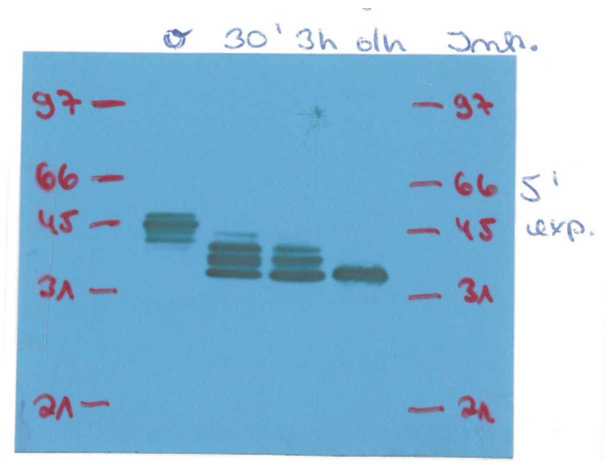


**Supplementary Figure 9. Uncropped version of the Western Blot shown in Figure 2c.** Analysis of the N-glycosylation pattern of sCASD1. Purified sCASD1-wt was analysed before (0) and after partial (30 min and 3 h) or complete (18 h, over night) Peptide-N-glycosidase F digest by 12% SDS-PAGE and Western blotting using an anti-Myc mAb.

a

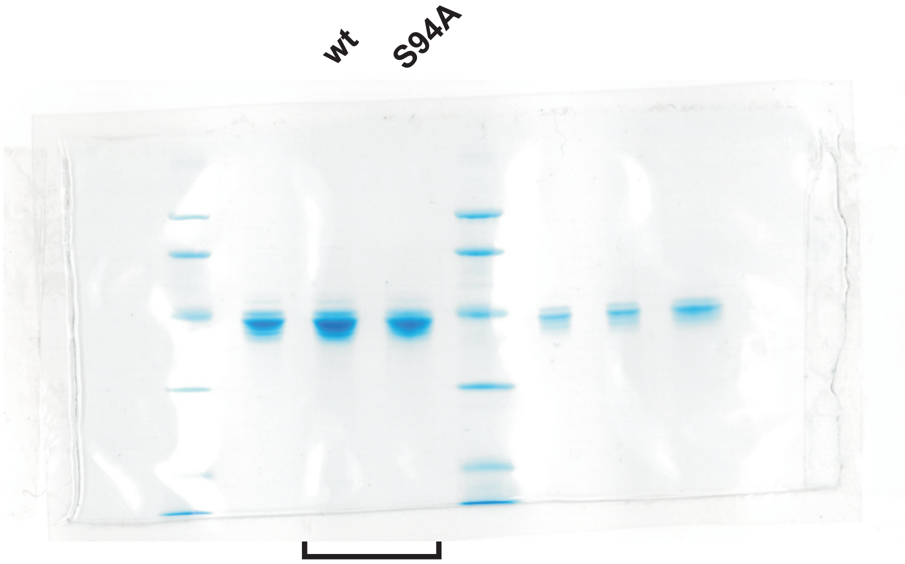


b

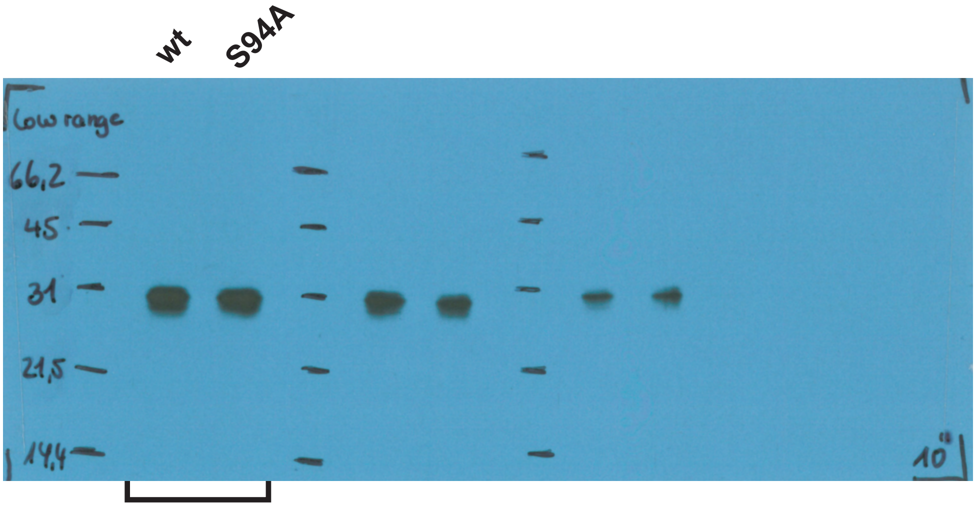


**Supplementary Figure 10. Uncropped versions of the gel and Western Blot shown in Figure 2b.** Soluble CASD1 variants encompassing the wild-type (wt) sequence or the amino acid exchange S94A were expressed in insect cells and affinity purified from the culture supernatant *via* the C-terminal hexa-histidine-tag. Purified proteins were analysed by 12% SDS-PAGE followed by Coomassie staining (**a**) or Western blotting using an anti-Myc mAb (**b**). Lanes shown in Figure 2b are highlighted by square brackets.

## Supplementary References


1. Krogh, A., Larsson, B., von Heijne, G. & Sonnhammer, E. L. Predicting transmembrane protein topology with a hidden Markov model: application to complete genomes. *J. Mol. Biol.* **305**, 567–80 (2001).
2. Carette, J. E. *et al.* Ebola virus entry requires the cholesterol transporter Niemann-Pick C1. *Nature* **477**, 340–343 (2011).
3. Feickert, H.J., Pietsch, T., Hadam, M.R., Mildenberger, H. & Riehm, H. Monoclonal antibody T-199 directed against human medulloblastoma: characterization of a new antigenic system expressed on neuroectodermal tumors and natural killer cells. *Cancer Res.* **49**, 4338–4343 (1989).
4. Chen, T. R., Dorotinsky, C., Macy, M. & Hay, R. Cell identity resolved. *Nature* **340**, 106 (1989).
5. Zimmer, G., Lottspeich, F., Maisner, A., Klenk, H.-D. & Herrler, G. Molecular characterization of gp40, a mucin-type glycoprotein from the apical plasma membrane of Madin-Darby canine kidney cells (type I). *Biochem. J.* **325**, 99–108 (1997).
6. Eckhardt, M. *et al.* Molecular characterization of eukaryotic polysialyltransferase-1. *Nature* **373**, 715–718 (1995).
7. Mühlenhoff, M., Manegold, A., Windfuhr, M., Gotza, B. & Gerardy-Schahn, R. The impact of N-glycosylation on the functions of polysialyltransferases. *J. Biol. Chem.* ***276***, 34066–34073 (2001).
8. Stanley, P. Glycosylation mutants of animal cells. *Annu. Rev. Genet.* **18**, 525–552 (1984).
9. Huang, J., Byrd, J. C., Yoon, W. H. & Kim, Y. S. Effect of benzyl-alpha-GalNAc, an inhibitor of mucin glycosylation, on cancer-associated antigens in human colon cancer cells. *Oncol. Res.* **4**, 507-515 (1992).
10. Huet, G. *et al.* Characterization of mucins and proteoglycans synthesized by a mucin-secreting HT-29 cell subpopulation. *J. Cell Sci.* **108**, 1275–1285 (1995).
11. Eckhardt, M., Mühlenhoff, M., Bethe, A. & Gerardy-Schahn, R. Expression cloning of the Golgi CMP-sialic acid transporter. *Proc. Natl. Acad. Sci.* **93**, 7572–7576 (1996).
12. Klein, A. *et al.* New sialic acids from biological sources identified by a comprehensive and sensitive approach: liquid chromatography-electrospray ionization-mass spectrometry (LC-ESI-MS) of SIA quinoxalinones. *Glycobiology* **7**, 421-432 (1997).
13. Zheng, Q. *et al*. Precise gene deletion and replacement using the CRISPR/Cas9 system in human cells. *BioTechniques* **57**, 115-124 (2014).
14. Kniep, B., Flegel, W. A., Northoff, H. & Rieber, E. P. CDw60 Glycolipid Antigens of Human Leukocytes: Structural Characterization and Cellular Distribution. *Blood* **82**, 1776–1786 (1993).
15. Schauer, R., Srinivasan, G. V., Wipfler, D., Kniep, B. & Schwartz-Albiez, R. *O*-Acetylated sialic acids and their role in immune defense. *Adv. Exp. Med. Biol.* **705**, 525–548 (2011).
16. Kniep, B. *et al.* 7-*O*-acetyl-GD3 in human T-lymphocytes is detected by a specific T-cell-activating monoclonal antibody. *J. Biol. Chem.* **270**, 30173–30180 (1995).
